# Amelioration of Alzheimer’s disease pathology by mitophagy inducers identified via machine learning and a cross-species workflow

**DOI:** 10.1038/s41551-021-00819-5

**Published:** 2022-01-06

**Authors:** Chenglong Xie, Xu-Xu Zhuang, Zhangming Niu, Ruixue Ai, Sofie Lautrup, Shuangjia Zheng, Yinghui Jiang, Ruiyu Han, Tanima Sen Gupta, Shuqin Cao, Maria Jose Lagartos-Donate, Cui-Zan Cai, Li-Ming Xie, Domenica Caponio, Wen-Wen Wang, Tomas Schmauck-Medina, Jianying Zhang, He-ling Wang, Guofeng Lou, Xianglu Xiao, Wenhua Zheng, Konstantinos Palikaras, Guang Yang, Kim A. Caldwell, Guy A. Caldwell, Han-Ming Shen, Hilde Nilsen, Jia-Hong Lu, Evandro F. Fang

**Affiliations:** 1 Department of Neurology, The First Affiliated Hospital of Wenzhou Medical University, Wenzhou, China; 2 Department of Clinical Molecular Biology, University of Oslo and Akershus University Hospital, Lørenskog, Norway; 3 Institute of Aging, Wenzhou Medical University, Wenzhou, China; 4 Oujiang Laboratory, Wenzhou, Zhejiang China; 5 Key Laboratory of Alzheimer’s Disease of Zhejiang Province, Wenzhou, China; 6 State Key Laboratory of Quality Research in Chinese Medicine, Institute of Chinese Medical Sciences, University of Macau, Macau, China; 7 Aladdin Healthcare Technologies Ltd., London, UK; 8 MindRank AI Ltd., Hangzhou, Zhejiang China; 9 School of Data and Computer Science, Sun Yat-sen University, Guangzhou, China; 10 Center of Traditional Chinese Medicine, The Second Affiliated Hospital and Yuying Children’s Hospital of Wenzhou Medical University, Wenzhou, China; 11 Faculty of Health Sciences, University of Macau, Taipa, Macau, China; 12 Department of Physiology, School of Medicine, National and Kapodistrian University of Athens, Athens, Greece; 13 Cardiovascular Research Centre, Royal Brompton Hospital, London, UK; 14 National Heart and Lung Institute, Imperial College London, London, UK; 15 Department of Biological Sciences, The University of Alabama, Tuscaloosa, AL USA; 16 Departments of Neurology and Neurobiology, Center for Neurodegeneration and Experimental Therapeutics, Nathan Shock Center for Research on the Basic Biology of Aging, University of Alabama at Birmingham School of Medicine, Birmingham, AL USA; 17 Department of Physiology, Yong Loo Lin School of Medicine, National University of Singapore, Singapore, Singapore; 18 Faculty of Health Sciences, University of Macau, Macau, China; 19 The Norwegian Centre on Healthy Ageing (NO-Age), Oslo, Norway; 20 Department of Geriatrics, The First Affiliated Hospital, Zhengzhou University, Zhengzhou, China

**Keywords:** High-throughput screening, Molecular medicine

## Abstract

A reduced removal of dysfunctional mitochondria is common to aging and age-related neurodegenerative pathologies such as Alzheimer’s disease (AD). Strategies for treating such impaired mitophagy would benefit from the identification of mitophagy modulators. Here we report the combined use of unsupervised machine learning (involving vector representations of molecular structures, pharmacophore fingerprinting and conformer fingerprinting) and a cross-species approach for the screening and experimental validation of new mitophagy-inducing compounds. From a library of naturally occurring compounds, the workflow allowed us to identify 18 small molecules, and among them two potent mitophagy inducers (Kaempferol and Rhapontigenin). In nematode and rodent models of AD, we show that both mitophagy inducers increased the survival and functionality of glutamatergic and cholinergic neurons, abrogated amyloid-β and tau pathologies, and improved the animals’ memory. Our findings suggest the existence of a conserved mechanism of memory loss across the AD models, this mechanism being mediated by defective mitophagy. The computational–experimental screening and validation workflow might help uncover potent mitophagy modulators that stimulate neuronal health and brain homeostasis.

## Abstract (web summary)

Two potent mitophagy inducers, identified and characterized via unsupervised machine learning and a cross-species screening approach, ameliorated the pathology of Alzheimer’s disease in worms and mice.

## Main

Accumulation of damaged mitochondria in the brain is a hallmark of brain aging and related neurodegenerative diseases, including Alzheimer’s disease (AD)^1–5^. As the most common form of dementia, AD affects around 50 million individuals worldwide without an available cure^6^. Accumulation of amyloid β (Aβ) and neurofibrillary tangles (majorly p-Tau aggregates) are the disease-defining pathological features of AD. However, clinical drug developments targeting Aβ and Tau have struggled to produce positive results^7^, highlighting the urgent need for discovery and development of novel therapeutic interventions. Mitochondria are fundamental subcellular organelles that generate adenosine triphosphate (ATP), which is essential for the excitability and survival of neurons. In addition, they are at the centre of signalling pathways regulating Ca^2+^, oxidative stress, developmental and synaptic plasticity, as well as neuronal fate determination^8^. Mitochondria constantly experience endogenous (for example, DNA damage and oxidative toxicants) and exogenous (for example, environmental exposure) stresses, which cause structural and/or functional damage to these essential organelles^9^. In a normal physiological environment, damaged mitochondria are efficiently cleared by mitophagy, a subtype of selective macroautophagy (hereafter referred to as autophagy)^10^. However, in elderly individuals or people with common neurodegenerative diseases such as AD, Parkinson’s disease, Amyotrophic lateral sclerosis and Huntington’s disease in which accumulation of defective mitochondria is a common feature, and possibly a driving force of memory impairment and dementia, autophagic processes may be disrupted^1,5,10^. Emerging evidence highlights that mitophagy impairment mediates the accrual of dysfunctional mitochondria in the AD brain^11^. Indeed, the basal levels of mitophagy are less than 50% in AD patient brain tissue compared with healthy controls^11^. Moreover, several regulators of autophagy and mitophagy pathways, such as phosphatidylinositol-binding clathrin assembly protein (PICALM)^12^, presenilin 1 (PS1)^13^, phosphatase and tensin homologue (PTEN)-induced kinase1 (PINK1), TANK-binding kinase 1 (TBK1), Unc-51-like kinase-1 (ULK1)^11^ and Bcl-2 associated athanogene 3 (BAG-3)^14^ are lowly expressed or impaired in AD patients. Genetic and/or pharmacologic restoration of mitophagy inhibits disease progression in preclinical AD models^11,15^. Given the continued failures in anti-AD drug development, approaches targeting the broader aspects of AD pathologies, such as defective mitophagy, may hold a therapeutic potential.

Bioavailable neuronal mitophagy inducers are scarce. Thus, we set out to develop a screening workflow combining advanced artificial intelligence (AI) and classical wet laboratory approaches to identify novel mitophagy modulators as potential drug candidates for AD treatment. The application of traditional chemistry or high-throughput approaches for drug discovery is time-consuming and also carry a high failure rate^16^. Machine learning is emerging as a powerful, fast, reliable and cost-effective approach to drug development, which can accelerate discovery and decision making for predefined questions with precise data^17–20^. Machine learning has been used in pharmaceutical development, bioactivity prediction, de novo molecular design, synthesis prediction and biological image analysis, among other applications^19,21,22^. It is a popular tool in drug discovery when using a large arsenal of compounds; however, the limitation of broad application is the necessity for a sizeable number of labelled data points to ensure model generalizability and avoid overfitting^19,22^. In view of the scarcity of known mitophagy inducers, an alternative machine learning approach is the use of ‘biological fingerprints’, which are representations of chemical structures originally designed to assist in chemical database substructure searching^23^. Here we outline the development of an AI-aided high-throughput screen workflow that combines AI, mammalian cells, nematodes and mice to create an approach for identifying potent mitophagy modulators.

## Results

### An AI-aided model for screening of mitophagy inducers

**Fig. 1:**
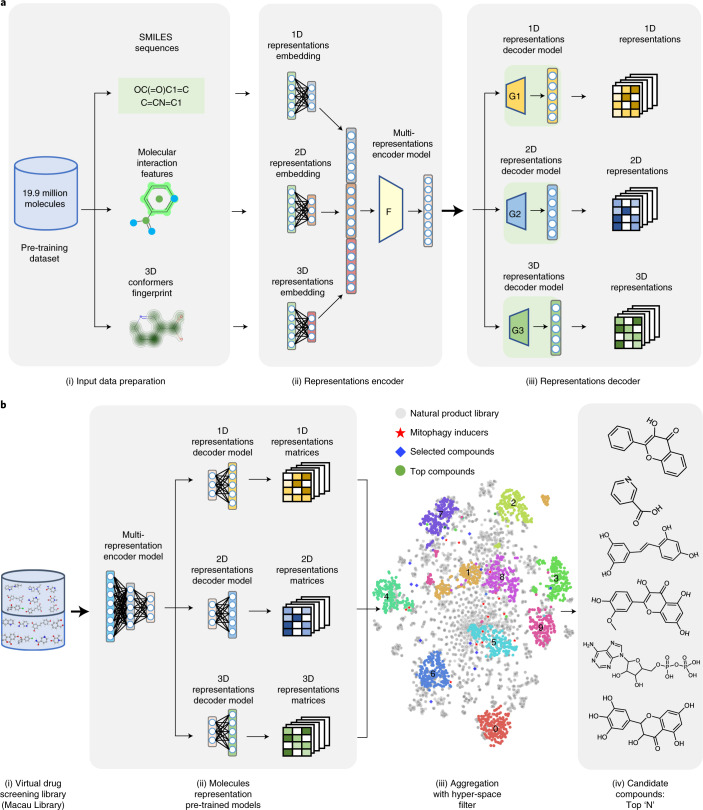
The use of combined machine learning strategies to identify novel mitophagy inducers. **a**, The workflow for model pre-training: (i) Molecules within the pre-training dataset were transferred into SMILES sequences, molecular interaction features and 3D conformers fingerprint in the data preparation stage; (ii) Three encoders (for 1D, 2D and 3D representations) were then designed to embed the input data, and these representational embeddings were aggregated into the encoder model of the multi-representation; (iii) The multi-representational embeddings were then passed to the representation decoder to pre-train the multi-representation molecule model. ‘F’ and ‘G’ stand for ‘Functional encoder’ and ‘Generator’ respectively. **b**, The workflow for the virtual screening process: (i) The virtual screening library contained 3,274 molecules from a traditional Chinese medicine dataset, named Macau Library; (ii) The 1D, 2D and 3D molecular representations for each compound were generated on the basis of the pre-trained molecule representation models; (iii) The representations were then aggregated and clustered, and a hyper-space filter was applied to the representations to filter out outliers; (iv) The similarity scores for each compound were calculated to generate the top N candidate compounds. Source data

A combinational molecular representation approach, including Mol2vec, pharmacophore fingerprint and 3D conformers fingerprint, was used for modelling (Fig. 1a,b). We first compiled a dataset that was large-scale, structurally diverse and task related. The ChEMBL^24^ and ZINC natural product databases^25^ were filtered using procedures outlined elsewhere^26^, producing a dataset with 19.9 million compounds (that is, the pre-training dataset). This pre-training dataset was used to train the multi-representations model, which translated a molecule into an information-enriched structure vector in an unsupervised manner, without the need for numerous annotated data. The model followed a natural language processing strategy^26^, wherein molecules were considered as sentences and substructures as words. By iteratively learning the relative position of each substructure in a molecule, the model could finally capture the global structural information of each substructure in the chemical space. New molecules could be described by summing the substructure vectors retrieved from a pre-trained Mol2vec model. The obtained compound feature vectors could then be used to calculate the structural distance of any two compounds in the projected chemical space. Further, to fill in the blanks of 2D pharmacophore and 3D conformer information, the pharmacophore and shape fingerprinting techniques were introduced to augment the representation of molecules (Fig. 1a). A total of 14 known mitophagy inducers were used as reference (Supplementary Table 1).

After model development and augmentation, we subsequently applied it to identify potential mitophagy inducers from a natural product library (named Macau Library), which contained 3,274 natural compounds isolated mainly from a series of traditional Chinese medicinal plants that have been used to treat neurodegenerative diseases and other diseases^27,28^. Similarity scores for each compound against each of the 14 known mitophagy inducers were determined, compounds were ranked on the basis of their structural (Mol2vec score), pharmacophore fingerprint and shape distance (3D conformers fingerprint) against the known inducers (Supplementary Table 1). We identified a total of 18 molecules in the Macau Library that were most similar to the existing (known) mitophagy inducers, with a threshold of 0.75. We set the threshold to 0.75 on the basis of published works^29,30^ and our in-house justification of the workload for wet-lab validation. Detailed information of the 18 in silico-selected molecules is documented (Supplementary Fig. 1 and Table 2). We further performed chemical similarity analysis of the top 18 molecules: while 1D similarities are lower than 40% between any two compounds, there are some compounds with high scores in 2D (for example, 91% for T2174 and T0579) and 3D (for example, 71% for T3S1068 and T2177) similarity analyses (Additional Supplementary Table). More details on the AI procedures can be found in Methods.

### In vitro and in vivo validation of mitophagy candidates

**Fig. 2:**
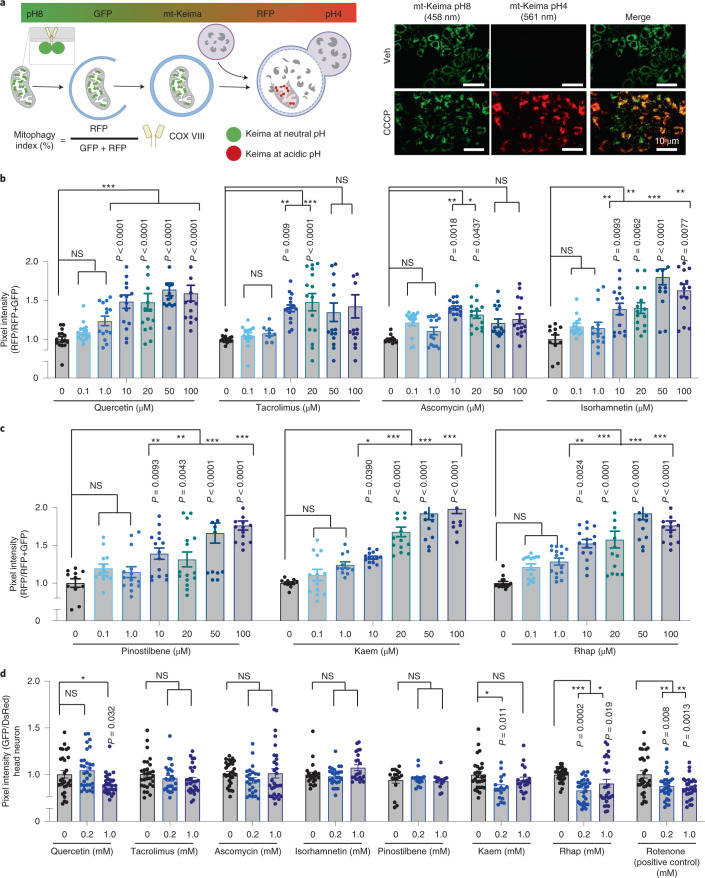
Evaluation of mitophagy stimulation capacity of the AI top-scored molecules in vitro (mt-Keima) and in animals (mt-Rosella). **a**, A schematic representation showing mechanisms of how the mt-Keima protein can be used as a mitophagy reporter. For confocal microscopy, dual-excitation ratio imaging was carried out with two sequential excitation lasers (458 nm and 561 nm). Representative confocal images are of HeLa cells expressing mt-Keima treated with vehicle (DMSO) or Carbonyl cyanide m-chlorophenyl hydrazone (CCCP) (15 μM, 3 h). **b**,**c**, Effects of Quercetin, Tacrolimus, Ascomycin, Isorhamnetin, Pinostilbene, Kaem and Rhap (from 0.1 μM to 100 μM, 24 h) on mitophagy induction. **d**, Effects of in vitro-positive mitophagy inducers on the induction of neuronal mitophagy in worms expressing mt-Rosella reporter. Rotenone (5 μM and 10 μM, 4 h) was used as positive control. Data were pooled from 2 biological replicates (total *n* = 20–35 nematodes per group), with results shown as mean ± s.e.m. Two-way ANOVA followed by Tukey’s multiple comparisons test; NS, no significance; *P < 0.05, **P < 0.01, ***P < 0.001. A set of representative images of cellular positive (related to Fig. 2b,c) and negative mitophagy inducers (with quantifications) is included in Supplementary Fig. 2. Mechanisms of the mt-Rosella sensor as well as a set of representative images (related to Fig. 2d) are shown in Supplementary Fig. 4. Source data

The 18 AI-selected molecules were then subjected to experimental verification in both human cells (HeLa cells) and the soil-dwelling nematode *Caenorhabditis elegans*. HeLa cells co-expressing the E3 ubiquitin ligase Parkin and the mitochondria-targeted form of monomeric Keima fluorescent reporter (mt-Keima)^31^ were used. Keima is a coral-derived, lysosomal degradation-resistant, dual-excitation ratio-metric fluorescent protein that is pH-sensitive; it shows shorter-wavelength excitation (green) in healthy mitochondria normally with neutral pH, while it turns to longer-wavelength excitation (red) in damaged mitochondria undergoing acidic lysosomal degradation (Fig. 2a)^31^. These features of the mt-Keima reporter allow qualitative assessment of mitophagic flux in both cells and mouse models^31–33^. To ensure high translational potential, we started with a series of doses covering 0.1, 1.0 and 10 μM, with 10 μM as the cut-off threshold. In a first-pass study of the 18 AI-selected compounds, 8 molecules (Quercetin (Macau Library ID: T-2174), Quercetin dihydrate (T-6630), Tacrolimus (T-2144), Ascomycin (T-2481), Isorhamnetin (T-2836), Pinostilbene (T-3755), Kaempferol (Kaem, T-2177) and Rhapontigenin (Rhap, T-3776)) induced mitophagy at 10 μM; the remaining 10 molecules did not induce detectable mitophagy up to 10 μM and were excluded from the study at this point (Fig. 2b,c and Supplementary Fig. 2a,c,d). It was noted that Quercetin dihydrate and Quercetin exhibited very similar results, likely due to their structural and functional similarities, thus Quercetin dihydrate was eliminated from the study at this point. To confirm whether the 7 remaining molecules trigger mitophagy in a dose-dependent manner, we administered higher doses of each compound (20, 50 and 100 μM) to the same HeLa mt-Keima cells. We were unable to observe any dose-dependent mitophagic upregulation in response to Quercetin, Tacrolimus and Ascomycin supplementation past 10 μM. However, Isorhamnetin, Pinostilbene, Kaem and Rhap administration triggered mitophagy in a dose-dependent manner (Fig. 2b,c). Therefore collectively, among the 18 AI-selected molecules, 8 showed an ability to stimulate mitophagy in vitro, with 4 of them inducing mitophagy in a dose-dependent manner.

To investigate whether the aforementioned mitophagy inducers could mediate neuronal mitophagy in vivo, we used transgenic nematodes with pan-neuronal expression of mitochondria-targeted Rosella (mt-Rosella, a dual colour-emission biosensor). The mt-Rosella biosensor comprises a green fluorescent protein (GFP) variant sensitive to the acidic environment of the lysosomal lumen, which is fused to the fast-maturing pH-insensitive DsRed. Mitophagy index is assessed by monitoring the GFP/DsRed ratio, with reduced values signifying mitophagy induction^11^ (Supplementary Fig. 4a). We supplemented 0.2 mM and 1.0 mM of each mitophagy-inducing compound to mt-Rosella-expressing animals from eggs onwards and analysed mitophagy levels in 1-day-old adults. Rotenone, a mitochondrial complex I inhibitor, was used as positive control to trigger mitophagy. Quercetin (at 1 mM), Kaem (at 0.2 mM) and Rhap (at both 0.2 mM and 1 mM) were able to induce neuronal mitophagy in worms, while Tacrolimus, Ascomycin, Isorhamnetin and Pinostilbene were negative for neuronal mitophagy induction (Fig. 2d and Supplementary Fig. 4b). In summary, among the 18 AI-selected candidates, 3 – Quercetin, Kaem and Rhap – stimulated mitophagy in both human cells and *C. elegans* neurons.

**Fig. 3:**
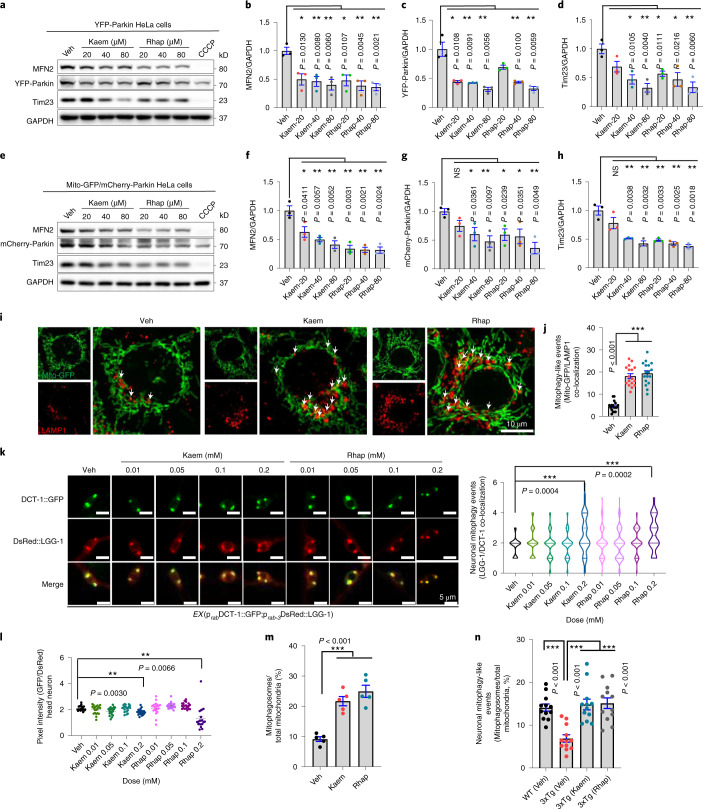
Kaem and Rhap induce mitophagy in cells, *C. elegans* neurons and mouse brain. **a**, Effects of Kaem and Rhap on the protein levels of mitofusin2 (MFN2), YFP-Parkin and Tim23 in HeLa cells stably overexpressing YFP-Parkin. **b**–**d**, Semi-quantification of **a** (*n* = 3 biological replicates). **e**, Effects of Kaem and Rhap on the protein levels of MFN2, YFP-Parkin and Tim23 in HeLa cells stably overexpressing Mito-GFP and mCherry-Parkin. **f**–**h**, Semi-quantification of **e** (*n* = 3 biological replicates). For **a** and **e**, CCCP was used as positive control. **i**, Images showing co-localization of mitochondria (Mito-GFP) and lysosomes (LAMP1 antibody) under Kaem and Rhap (20 uM, 24 h) administration in GFP-mito-mCherry-Parkin HeLa cells. White arrows indicate mitophagy events. **j**, Quantification of **i** with data from 3 biological repeats with around 5–7 images per biological repeat. **k**, Transgenic nematodes were treated with Kaem and Rhap (both with 0.01, 0.05, 0.1 and 0.2 mM), with mitophagy events calculated by the co-localization between the autophagic marker DsRed::LGG-1 and the mitophagy receptor DCT-1::GFP in neurons. *n* = 18–20 neurons from 2 biological repeats. While the left panel shows one representative set of images, quantitative data are shown in the right panel. **l**, Effects of Kaem and Rhap on the induction of neuronal mitophagy in worms with mt-Rosella reporter. Data were pooled from 2 biological replicates (total *n* = 20–35 nematodes per group), with the results shown as mean ± s.e.m. **m**,**n**, Data of quantified electron microscopic images showing effects of Kaem and Rhap on mitochondrial morphology and mitophagy-like events in mt-Keima HeLa cells (**m**) (20 µM for 24 h) and mouse hippocampal brain tissues (**n**) (100 mg kg^−1^ d^−1^ via oral gavage from 12 months for 7 consecutive days; *n* = 3 mice per group, with 4 random hippocampal neuronal images per mouse). Representative images are shown in Extended Data Fig. 1a,b. All quantitative data are shown as mean ± s.e.m. One-way ANOVA followed by Šidák’s multiple comparisons test; **P 0.01, ***P 0.001. Original unprocessed western blot gel data are in Source Data Fig. 4. Source data

**Extended Data Fig. 1:**
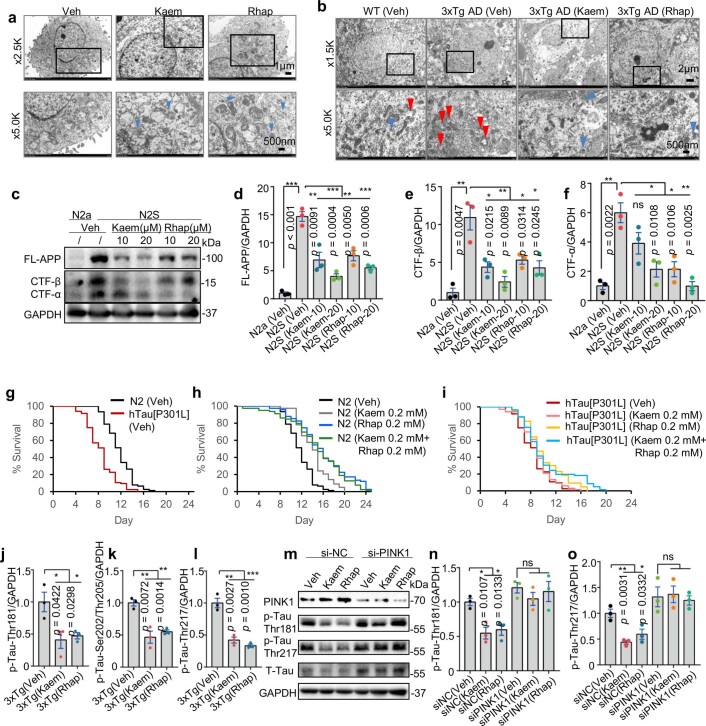
Kaem and Rhap induce mitophagy, abrogate disease pathologies, and improve healthspan and lifespan in cells, worm, or mouse models of AD. **a**, **b**, electron microscopic images showing effects of Kaem and Rhap on mitochondrial morphology and mitophagy-like events in mt-Keima HeLa cells (a, 20 µM for 24 h) and mouse hippocampal brain tissues (**b**, 100 mg/kg/d via oral gavage from 12 months for 7 consecutive days; n = 3 mice per group with 4 random hippocampal neuronal images per mouse), treated with Kaem and Rhap. Blue arrows indicate mitophagosome-like events, while red arrows point to damaged mitochondria. Quantitative data of a and b are shown in Fig. 3m and Fig. 3n, respectively. **c**–**f**, Effects of Kaem and Rhap on the expression of designated proteins involved in Aβ production. N2a and N2S (N2a stably transfected with human Swedish mutant APP695) cells were used. One set of western blot data is shown (**c**), with semi-quantifications from three biological replicates (**d**–**f**). All quantitative data were shown in mean ± S.E.M. One-way ANOVA followed by Šidák’s multiple comparisons test (**d**-**f**) were used for data analysis. NS, no significance, *p < 0.05, **p < 0.01, ***p < 0.001. Original western blot gels for (**c**) are included in Source Data Fig. 2. **g**–**i**, Effects of Kaem and Rhap on lifespan of the N2 and hTau[P301L] (CK12) worms. Data shown are from one set of experiments from a total of two biological replicates (quantitative values in Supplementary Table 4). 90–120 worms were used for each group/biological repeat. All quantitative data were shown in mean ± S.E.M. Log-rank test was used for the statistics of lifespan data (**g**–**i**). NS, no significance, *p < 0.05, **p < 0.01, ***p < 0.001. **j**–**l**, Quantification of phosphorylated Tau sites (Thr181, Ser202/Thr205, Thr217) in hippocampal tissues from the 3xTg AD mice (n = 3 biological replicates). The western blotting gel data are shown in Fig. 6o. **m**–**o**, Effects of *PINK1* knockdown on p-Tau inhibition by Kaem and Rhap in HEK 293 3G-EGFP-Tau P301L/mCherry cells. While one biological set of blot data are shown (**m**), quantifications (**n**, **o**) were from 3 biological replicates. All quantitative data were shown in mean ± S.E.M. Two-way ANOVA followed by Tukey’s multiple comparisons test (**j, k**. **l**) and one-way ANOVA followed by Šidák’s multiple comparisons test (**n**, **o**) were used for data analysis. NS, no significance, *p < 0.05, **p < 0.01, ***p < 0.001. Original western blot gels for (**m**) were included in Source Data Fig. 2.

In addition to using HeLa mt-Keima cells and mt-Rosella-expressing animals to quantify mitophagy by Kaem and Rhap, we further validated the robust mitophagy induction capacities of Kaem and Rhap. Firstly, immunoblot data indicate that both Kaem and Rhap dose-independently (20, 40 and 80 μM for 24 h) reduced the expression of the mitochondrial outer membrane protein MFN2 and mitochondrial inner membrane protein Tim23 in both YFP-Parkin-expressing HeLa cells and Mito-GFP- and mCherry-Parkin-expressing HeLa cells (Fig. 3a–h). Secondly, Kaem and Rhap (20 μM, 24 h) enhanced co-localization of mitochondria (Mito-GFP) with the LAMP1-antibody-labelled lysosome, indicating increased lysosomal degradation of mitochondria via mitophagy (Fig. 3i,j). Thirdly, Kaem and Rhap at 0.2 mM stimulated neuronal mitophagy in *C. elegans* as evidenced by increased LGG-1/Atg-8 to DCT-1/NIX co-localization, and increased mitochondria in the lysosomes as shown by reduced GFP/DsRed (Fig. 3k,l). Fourthly, data from electron microscopy (EM) showed that Kaem and Rhap induced mitophagosome-like events in HeLa cells (Fig. 3m and Extended Data Fig. 1a), as well as in hippocampal brain tissues from wild-type (WT) and AD-like 3xTg mice (Fig. 3n and Extended Data Fig. 1b; details on the mouse studies are shown below). To note, lower doses of either Kaem or Rhap were unable to induce mitophagy in HeLa mt-Keima cells (2.5 and 5 µM, 24 h; Supplementary Fig. 2b) or the nematode neurons (0.01, 0.05, 0.1 mM; Fig. 3k,l). Collectively, these data unequivocally point to robust mitophagy stimulation capacity of both Kaem and Rhap in cell culture system, nematodes and mice.

Additionally, we compared our combinational AI model (Fig. 1a) with other machine learning approaches (1D, 2D or 3D) to determine their accuracy in identifying mitophagy inducers. We selected the top 5 scored compounds from each of the independent approaches for validation in HeLa mt-Keima cells. Compounds in the 1D- and 3D-selected lists, at 10 µM, were unable to induce detectable mitophagy in HeLa mt-Keima cells (Supplementary Fig. 3). All top 5 compounds recommended by the 2D approach were in the top 18 list selected by our combinational AI model: 3 compounds (T0879, T2812, T2144 at 10 µM) were unable to induce mitophagy, the remaining 2 (Ascomycin (T2481) and Pinostilbene (T3755)) were able to induce mitophagy in cells, but were unable to induce neuronal mitophagy in nematodes (Fig. 2). Additionally, we reviewed and listed the hit rate for experimental high-throughput screening and other AI drug discovery projects for a comprehensive comparison study. Overall, the hit rate of our model (in vitro validation) is higher than the experimental high-throughput screening (44% vs 0.14%) and substantially outperforms other machine learning, quantitative structure–activity relationship (QSAR) and computer-aided approaches (Supplementary Table 3). Collectively, these in silico, in vitro and in vivo data indicate that our combinational AI approach is more accurate in predicting molecules with mitophagy induction and neuroprotection activities both in vitro and in vivo, than the individual 1D, 2D or 3D approaches.

### Kaem and Rhap inhibit memory loss in Aβ_1–42_*C. elegans*

**Fig. 4:**
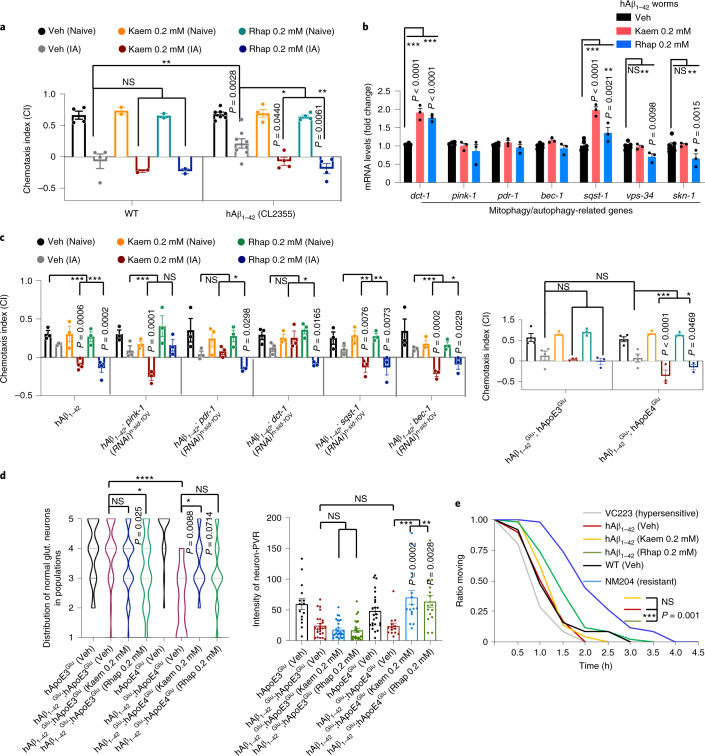
Mitophagy stimulation restores memory deficit and abrogates pathologies in AD-like Aβ worms, and regulates cellular Aβ production in mouse neuroblastoma cells. **a**, Effects of Kaem and Rhap on associative memory in adult day 1 WT and hAβ_1–42_ (CL2355) worms. Data were pooled from at least 4 biological replicates. **b**, Effects of Kaem and Rhap on designated gene expression in day 1 adult worms. Data are from 1 representative biological repeat (3 technical repeats) from a total of 3 biological replicates. **c**, Left: effects of *pink-1*, *pdr-1*, *dct-1*, *sqst-1* and *bec-1* on Kaem- and Rhap-dependent memory improvement in the hAβ_1–42_ (CL2355) worms. Right: effects of Kaem and Rhap on associative memory in adult day 1 hAβ_1–42_^Glu^;hApoE3^Glu^ (UA353) and hAβ_1–42_^Glu^;hApoE4^Glu^ (UA355) worms. ‘^Glu’^ denotes that either hAβ_1–42_ or hApoEs were expressed only in the glutamatergic neurons. Data were pooled from at least 4 biological replicates. **d**, Effect of Kaem or Rhap on glutamatergic neuroprotection in the hAβ_1–42_^Glu^;ApoE4 worms and other worm strains. Left: distribution of worms with different numbers of 5 designated tail neurons (*n* = 80–100 from 2 biological replicates). Right: the fluorescent intensity of PVR neurons (*n* = 15 from 2 biological replicates). **e**, Effects of Kaem and Rhap on acetylcholinesterase inhibitor aldicarb-induced paralysis. VC223 (a strain hypersensitive to aldicarb-induced paralysis) and NM204 (a strain resistant to aldicarb-induced paralysis) were used as controls. All quantitative data are shown as mean ± s.e.m. Two-way ANOVA followed by Tukey’s multiple comparisons test (**a**–**e**); NS, no significance; *P < 0.05, **P < 0.01, ***P < 0.001. Effects of Kaem and Rhap on Aβ generation in mouse neuroblastoma cells are shown in Extended Data Fig. 1c–f. Additional data related to **e** are in Supplementary Fig. 6. Source data

Recent evidence underlies the likely causative role of compromised mitophagy in AD pathogenesis^11,34^. Thus, we examined the impact of the newly identified mitophagy stimulators on memory improvement in both Aβ and Tau nematode models. To investigate whether pharmacological upregulation of mitophagy restores memory deficits, we evaluated learned behaviour in transgenic nematodes, whereby they have pan-neuronal expression of human Aβ_1–42_ (hAβ_1–42_)^35^, via aversive olfactory learning chemotaxis assay (where a negative value correlates with chemotaxis-related memory)^11^. hAβ_1–42_ nematodes treated with Kaem or Rhap displayed improved learned behavioural performance, while Quercetin did not appear to restore associative memory deficits (Fig. 4a and Supplementary Fig. 5a). Thus, among the 18 AI-selected candidates, Kaem and Rhap demonstrated the capacity to stimulate mitophagy in both human cells and *C. elegans* neurons, and improved an established measure of simple associative memory in these transgenic hAβ_1–42_ worms.

**Extended Data Fig. 2:**
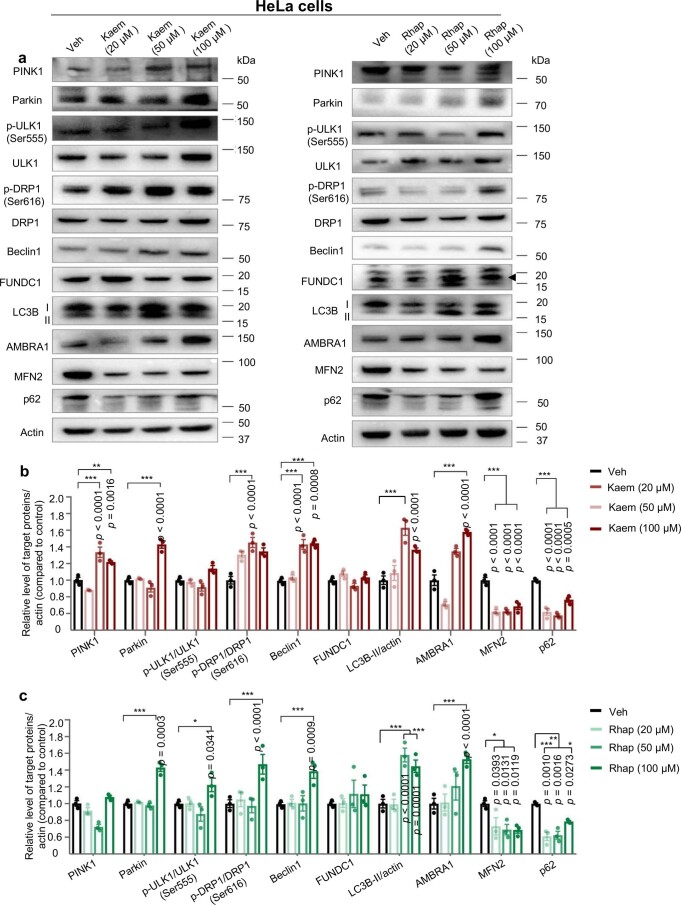
Kaem and Rhap induce expression of a broad spectrum of autophagy/mitophagy proteins. **a**, A representative set of western blot images for designated proteins upon Veh., Kaem (20, 50, 100 μM) or Rhap (20, 50, 100 μM) administration for 24 h. Arrow head in the right panel pointed to ‘FUNDC1’, with bands upper and lower were likely unknown or non-specific bands. **b**, **c**, Semi-quantification of **a** from three biological replicates with data shown in mean ± S.E.M. One-way ANOVA followed by Šidák’s multiple comparisons test was used for data analysis, with *p < 0.05, **p < 0.01, ***p < 0.001. Original western blot gels for (**a**) were included in Source Data Fig. 1.

We then investigated the underlying molecular mechanisms to identify how Kaem and Rhap improve memory, focusing on mitophagy/autophagy-related pathways. While none of the compounds had any effect on the mRNA levels of *pink-1*, *pdr-1* (orthologue of human *PARK2/Parkin)*, *bec-1* (orthologue of human *BECN1/Beclin-1*), *vps-34* and *skn-1* (a stress response gene also involved in mitophagy^36^), they both increased transcriptional levels of *dct-1* and *sqst-1* (*SQSTM1* gene in humans) in transgenic hAβ_1–42_ worms (Fig. 4b). To consider translational and post-translational modifications, and due to limitations in available antibodies for *C. elegans* studies, we extended our mechanistic studies to human HeLa cells and scrutinized the mitophagy-inducing capacity of the molecules by checking them against a list of mitophagy/autophagy proteins that are known to be critical in mitochondrial metabolism or that are altered in AD^11^. In most cases, Kaem increased levels of PINK1, Parkin, Beclin-1, LC3B-II and AMBRA1, and reduced p62 in a dose-dependent manner; a very similar pattern was seen in the Rhap-treated human cells (Extended Data Fig. 2). Moreover, Rhap treatment increased the phosphorylation levels of the autophagy factor ULK1 at Ser555, which is essential for mitophagy initiation^37^ (Extended Data Fig. 2). In addition, to increase the expression of multiple mitophagy-related proteins, Kaem or Rhap supplementation resulted in reduced protein levels of Mitofusin-2 (MFN2) and elevated the ratio of p-DRP1 (Ser616)/DRP1 (Extended Data Fig. 2). These findings suggest that mitochondrial fission is triggered upon Kaem and Rhap treatment, leading to the generation of smaller and fragmented organelles that can be easily engulfed by autophagosomes^38^. To further investigate whether Kaem- and Rhap-dependent memory improvement in the hAβ_1–42_ worms is due to mitophagy induction (rather than any off-target effects), we introduced loss-of-function mutations (*pink-1*, *pdr-1*, *dct-1*) or knocked down selected genes (*sqst-1*, *bec-1*, via RNAi) on the basis of PCR and immunoblot data. Depletion of *pdr-1* and *dct-1* abrogated Kaem-dependent memory improvement in the hAβ_1–42_ worms, whereas Rhap-induced memory improvement was dependent on the *pink-1* pathway (Fig. 4c(left) and Supplementary Fig. 5f). Collectively, Kaem and Rhap appear to trigger mitophagy through the upregulation and/or activation of specific mitophagy components and via the modulation of mitochondrial dynamics.

To uncover the mechanisms of memory retention mediated by Kaem and Rhap at a cellular level, we asked whether these compounds act through the glutamatergic or cholinergic neurons, which are both impaired in AD^39^. Overexpression of hAβ_1–42_ specifically in the glutamatergic neurons in worms (hAβ_1–42_^Glu^) has been shown to induce neurodegeneration^36,40^. We examined whether Kaem and Rhap could alleviate neurodegeneration and improve associative learning in these hAβ_1–42_^Glu^ worms. Both Kaem and Rhap administered at 0.2 mM improved memory deficits observed in hAβ_1–42_^Glu^; hApoE4^Glu^ worms, as evidenced by the restoration of the chemotactic index to a level even surpassing that of hAβ_1–42_^Glu^; hApoE3^Glu^ control animals (Fig. 4c(right)). As previously described^35,36^, five specific glutamatergic neurons in the tail region of *C. elegans* hermaphrodites (LUA (R), LUA (L), PVR, PLM (R) and PLM (L)) (Supplementary Fig. 5g(left) and Video) afford a means of robust quantification of neurodegeneration in vivo. These anatomically isolated neurons facilitate unparalleled accuracy in scoring loss of neuronal processes and cell bodies at the single-neuron level, as they reproducibly degenerate in response to constitutive hAβ expression, and this occurs progressively with age^40,41^. Kaem inhibited glutamatergic neurodegeneration in adult Day 3 hAβ_1–42_^Glu^; hApoE4^Glu^ worms (Fig. 4d(left)). There was a trend towards glutamatergic neuroprotection by Rhap as a small percentage of worms reached 5 neurons, with fewer worms in the 1–2 neurons groups (Fig. 4d(left); *P* = 0.0714). Indeed, in addition to inhibiting neuronal loss, administration of either Kaem or Rhap improved neuronal health as evidenced by increased GFP intensity (for example, in PVR neurons, Fig. 4d(right)) and also improved neuronal morphology (Supplementary Fig. 5g). Impairment of the cholinergic system plays an important role in the pathophysiology of AD, and cholinergic therapies serve as a standard pharmacological approach in AD^42^.To investigate the effect of Kaem or Rhap on synaptic transmission of acetylcholine, pan-neuronal hAβ_1–42_ worms were exposed to an acetylcholinesterase inhibitor, aldicarb, and the time-course of paralyzing effects was scored^43^. While Kaem had no detectable effect on aldicarb-induced paralysis, Rhap greatly improved resistance of the hAβ_1–42_ worms to aldicarb-induced paralysis, indicating possible cholinergic protection by Rhap (Fig. 4e and Supplementary Fig. 6a,b). The aldicarb-hypersensitive strain VC223 and the aldicarb-resistant NM204 were used as controls (Supplementary Fig. 6g).

We further asked whether Kaem and Rhap could reduce hAβ_1–42_ production by using N2a mouse neuroblastoma cells expressing the Swedish K595N and M596L mutations in Amyloid-beta precursor protein/APP (APPSwe). Both Kaem and Rhap reduced full-length APP, CTF-α (non-amyloidogenic pathway via α-secretase) and CTF-β (amyloidogenic pathway via β-secretase) in these mouse neuroblastoma cells in a dose-dependent manner, suggesting a possibility of reduction in amounts of CTF-β available to be cleaved to hAβ_1–42_ via γ-secretase (Extended Data Fig. 1c–f, and further verified in mouse brain detailed below). In summary, Kaem and Rhap improve the function or survival of glutamatergic and cholinergic neurons and reduce hAβ_1–42_ production.

### Kaem and Rhap improve memory by reducing Tau pathologies

**Fig. 5:**
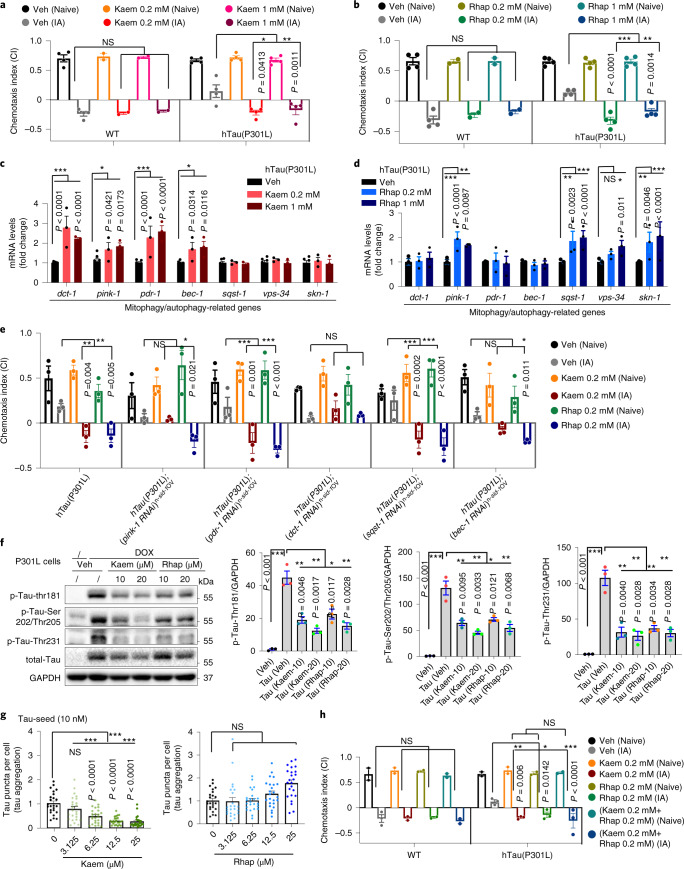
Mitophagy stimulation restores memory deficit in the AD-like hTau(P301L) *C. elegans* model and inhibits Tau pathologies in mammalian cells. **a**,**b**, Effects of Kaem (**a**) or Rhap (**b**) on associative memory in transgenic nematodes expressing hTau(P301L) (CK12). Data were pooled from 4 biological replicates. **c**,**d**, Effects of Kaem (**c**) and Rhap (**d**) on designated gene expressions in adult day 1 worms. Data are from a total of 3 biological replicates. **e**, Effects of *pink-1*, *pdr-1*, *dct-1*, *sqst-1* and *bec-1* on Kaem- and Rhap-dependent memory improvement in the hTau(P301L) worms. **f**, Western blot data with semi-quantifications showing changes in designated phosphorylated Tau sites in the HEK 293 cells expressing pTRE3G-mcherry-BI promoter-EGFP Tau P301L (HEK 293 3G-EGFP-Tau P301L/mCherry) with 24 h treatment of Kaem or Rhap. **g**, Effects of Kaem and Rhap on seeded Tau-induced endogenous Tau aggregation in the HEK293 cells expressing 0N4R P301S Tau-Venus. Data are from 3 biological replicates. **h**, Evaluation of any synergistic effects of Kaem and Rhap on associative memory in hTau(P301L) (CK12) worms. Data were pooled from 3 biological replicates. All quantitative data are shown as mean ± s.e.m. Two-way ANOVA followed by Tukey’s multiple comparisons test (**a**–**h**). NS, no significance; *P < 0.05, **P < 0.01, ***P < 0.001. Additional Tau seeding data are included in Extended Data Fig. 3h–k. Original western blot gels for **f** are included in Source Data Fig. 2.

In addition to alleviating Aβ pathology, we asked whether Kaem and Rhap protect against tauopathies by using the well-characterized CK12 (hTau4R1N(P301L)^44^, pan-neuronal expression) and BR5270 (with pro-aggregant hTau(F3Δ280) tau fragment^45^, pan-neuronal expression) strains. While the hTau(P301L) worms are known to have impaired memory^11^, both Kaem and Rhap improved memory in this Tau strain (Fig. 5a,b). Tacrolimus, Ascomycin, Isorhamnetin or Pinostilbene did not show associative memory improvement to the hTau(P301L) worms at 1 mM, but Tacrolimus and Isorhamnetin improved associative memory at 0.2 mM (Supplementary Fig. 5b–e).

**Extended Data Fig. 3:**
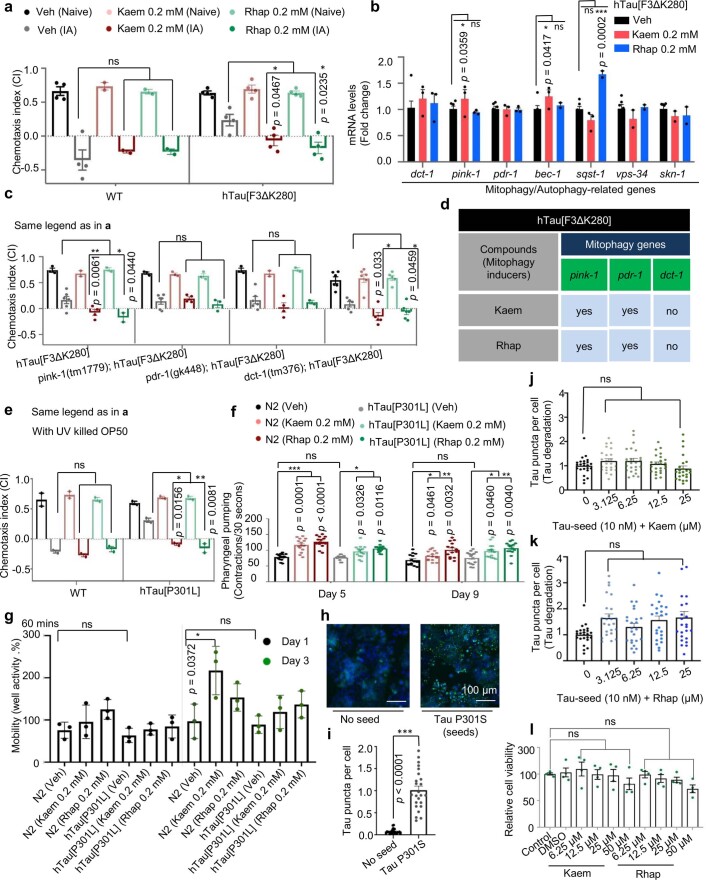
Mitophagy stimulation restores memory deficit and improves healthspan in the AD-like hTau[F3ΔK280] *C. elegans* model. **a**, Effects of Kaem and Rhap on associative memory in transgenic animals expressing hTau[F3ΔK280]. **b**, mRNA levels of genes affected by Kaem and Rhap in the hTau[F3ΔK280] worms. **c**, Effects of *pink-1*, *pdr-1*, *dct-1, sqst-1, and bec-1* on Kaem- and Rhap-induced associative memory in the hTau[F3ΔK280] worms. **d**, A summary of mitophagy genes involved in Kaem and Rhap-induced memory improvement in the hTau[F3ΔK280] worms. **e**, Memory assay of worms maintained on UV-killed OP50 treated with 0.2 mM Kaem, 0.2 mM Rhap or Veh. **f**, Pharyngeal pumping rates at days 1, 5 and 9 of adulthood in worms treated with 0.2 mM Kaem, 0.2 mM Rhap or Veh (n = 15). **g**, Mobility of hTau[P301L] worms treated with Kaem (n = 50–100 worms) or Rhap (n = 50–100 worms). Adult Day-1 worms were used. One set of data are shown from a total of two biological replicates. **h**–**k**, Effects of Kaem and Rhap on reduction of aggregated Tau in the HEK293 P301S Tau-Venus cells. Exogenous recombinant heparin-assembled P301S Tau (Tau seeds) induces conversion of endogenous Tau Venus from a dispersed distribution to bright foci (h, i). Tests to determine the mechanism of the effect of drugs affecting the degradation of aggregated Tau were performed (**j**, **k**). One image/well was taken, with a total of 8 technical repeats per biological replicate. Data were analysed using ImageJ with images from 3 biological replicates. **l**, Toxicity evaluation of Kaem or Rhap on PC12 cells using MTT. All quantitative data are shown in mean ± S.E.M. Two-way ANOVA followed by Tukey’s multiple comparisons test (**a**–**c**, **e**–**g**), one-way ANOVA followed by Šidák’s multiple comparisons test (**j**–**l**), and student *t*-test (**i**) were used for data analysis. NS, no significance, *p < 0.05, **p < 0.01, ***p < 0.001.

Kaem increased mRNA levels of *dct-1*, *pink-1*, *pdr-1* and *bec-1*, while having no effect on *sqst-1*, *vps-34* or *skn-1* in the hTau(P301L) worms (Fig. 5c). Rhap treatment resulted in increased mRNA levels of *pink-1*, *sqst-1*, *vps-34* and *skn-1* without having an effect on *dct-1*, *pdr-1* or *bec-1* (Fig. 5d). Kaem-induced memory improvement in hTau(P301L) worms was in a *pink-1*-*, dct-1*- and *bec-1*-dependent manner; Rhap-induced memory improvement in hTau(P301L) worms was in a *dct-1-*dependent manner (Fig. 5e). Furthermore, both Kaem and Rhap greatly improved the resistance of the hTau(P301L) worms against aldicarb-induced paralysis, indicating possible cholinergic protection (Supplementary Fig. 6c,d). Further investigation into the effects of Kaem and Rhap in hTau(F3Δ280) worms showed that both Kaem and Rhap improved the memory capacity in these worms^11^ (Extended Data Fig. 3a). Mechanistically, although Kaem and Rhap had no or only minor changes in *pink-1* and *pdr-1* mRNA levels (Extended Data Fig. 3b), Kaem- and Rhap-induced memory improvement in the hTau(F3Δ280) worms was in a *pink-1*- and *pdr-1*-, but not *dct-1*-dependent manner (Extended Data Fig. 3c,d). Furthermore, both Kaem and Rhap greatly improved the resistance of the hTau(F3Δ280) worms against aldicarb-induced paralysis, indicating possible cholinergic protection (Supplementary Fig. 6e,f). In addition to memory benefit, Kaem and Rhap also improved healthspan measured as an enhancement of pharyngeal pumping in the hTau(P301L) worms (Extended Data Fig. 3f), although no statistical significance in improved mobility was observed (Extended Data Fig. 3g).

We then examined whether Kaem or Rhap supplementation exerts a beneficial effect by reducing pathological Tau aggregates. Phosphorylations of designated Tau sites are essential for Tau aggregation. Indeed, Kaem and Rhap diminished Tau phosphorylation levels at multiple sites such as Thr181, Ser202/Thr205 and Thr231 in mammalian HEK293-Tau P301L cells (Fig. 5f). Considering that Tau fibrils mediate transmission of neurofibrillary tangles (a possible hypothesis of age-dependent spreading of Tau pathology in AD patients^46–48^), we asked whether Kaem and Rhap could alleviate ‘seeded Tau’-induced Tau pathology. To explore this angle, we employed a high-content microscopy-based assay using HEK293 cells stably expressing the 0N4R isoform of human Tau, bearing the P301S mutation with a C-terminal Venus fluorescent protein tag^49^. It has been reported that ectopic addition of recombinant heparin-assembled P301S Tau (Tau seeds with no Venus tag) promoted the generation of intracellular bright Tau foci from a disperse distribution, indicating extracellular inclusion of pathological Tau seed-induced intracellular Tau aggregation^49^ (Extended Data Fig. 3h,i). While Rhap had no statistically significant effect on the reduction of Tau seed-induced intracellular Tau aggregation from a dose range between 3.125 µM to 25 µM, Kaem dramatically reduced intracellular Tau aggregation in a dose-dependent manner, with 25 µM showing over 80% reduction (Fig. 5g). Moreover, we assessed whether Kaem or Rhap affects the degradation of Tau aggregates; however, both did not reduce the numbers of Tau foci at up to 25 μM for 24 h (Extended Data Fig. 3j,k). Kaem-induced decrease of seed-induced Tau aggregation was not due to cell death, as we did not detect any side effect on cell viability even up to 50 μM of Kaem for 24 h (Extended Data Fig. 3l). To conclude, Kaem and Rhap improve memory in Tau-expressing nematodes, as well as antagonize multiple p-Tau sites and/or Tau aggregation.

### Effects of combined treatment and other factors on the hTau worms

We further investigated whether a combination of Kaem and Rhap had any additive or synergistic effect to combat memory loss in the AD worms. In the transgenic hTau(P301L) worms, a dose of either 0.2 mM Kaem or Rhap was able to improve memory. When administered together, we did not observe any additive or synergistic benefit in memory improvement (Fig. 5h). Further, since population studies indicate that individuals with AD have a shorter lifespan than healthy controls^50,51^, we examined whether enhanced memory (Fig. 5a,b) and healthspan (Extended Data Fig. 3f) also correlated with an increased lifespan. While the hTau(P301L) worms had a shorter lifespan compared with the WT N2 worms (Extended Data Fig. 1g), Kaem (0.2 mM) or Rhap (0.2 mM) administration extended the WT N2 nematode average lifespan by 24.5% and 32.5%, and hTau(P301L) nematode lifespan by 5.8% and 17.4%, respectively (Extended Data Fig. 1h,i and Supplementary Table 4). No synergistic effects in lifespan extension were noticed when combining Kaem (0.2 mM) and Rhap (0.2 mM) in either hTau(P301L) worms or N2 control animals (Extended Data Fig. 1h,i and Supplementary Table 4).

The changes in the biological effects (for example, chemotaxis-based memory performance) on the worms as induced by the tested compounds may also be attributable to alternate mechanisms, such as microbial metabolism (for example, dependence on the live food *Escherichia coli* (OP50) to metabolize the metabolic precursors to more bioactive ones) or changes in eating pattern due to a dietary restriction mimetic^52,53^. We performed additional analyses looking into the potential effect of microbial metabolism and eating habits on memory. Since both Kaem and Rhap were supplemented in the hTau(F3Δ280)-expressing worms fed dead OP50, memory improvements were not likely to have been affected by microbial metabolism (Extended Data Fig. 3e). Likewise, exposing the worms to Kaem and Rhap likely did not reduce short-term food intake, but rather increased it; this may be due to the increased pharyngeal pumping observed with drug treatment (Extended Data Fig. 3f), thus excluding any indirect effects arising from dietary restriction.

### Kaem and Rhap forestall pathologies in the 3xTg AD mice

**Fig. 6:**
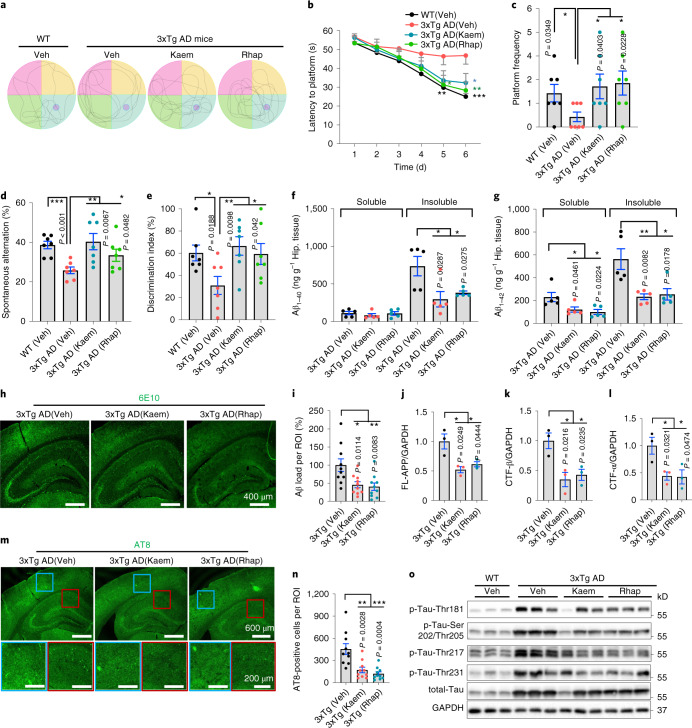
Mitophagy stimulation forestalls memory loss and ameliorates both Aβ and Tau pathologies in 3xTg AD mice. The 3xTg AD mice were treated with Kaem or Rhap (100 mg kg^−1^ d^−1^) via oral gavage for 2 months starting from 12.5 months of age. **a**, Representative images of the swimming tracks of mice at day 7 in the Morris water maze test (*n* = 6 mice per group). **b**, Latency to the platform of mice from days 1 to 6. **c**, Platform frequency of mice in the probe trial at day 7. **d**,**e**, Effects of Kaem and Rhap on spontaneous alternation (**d**) (Y maze) and novel object recognition/NOR (**e**). **f**,**g**, Soluble and insoluble Aβ_1–40_ and Aβ_1–42_ levels in hippocampal tissues. *n* = 5 mice in all groups. **h**, Immunohistochemical analysis of Aβ load in 3xTg AD mice hippocampi and cortices under Kaem or Rhap treatment. Experiments were repeated twice independently, with similar results. **i**, Quantification of Aβ load per ROI in images from **h**. *n* = 10 random areas in the ROIs from 3 mice per group. **j**–**l**, Semi-quantification of western blot data showing effects of Kaem and Rhap on the levels of full-length APP (FL-APP), CTF-β and CTF-α in hippocampal tissues from the 3xTg AD mice (*n* = 3 biologically independent samples). **m**, Representative immunofluorescence staining of AT8-positive cells in the cortex and hippocampus of 3xTg AD mouse brains. Experiments were repeated twice independently, with similar results. The blue and red squares denote designated brain regions were magnified. **n**, Quantified data of **m** (*n* = 10 random areas in the ROIs from 3 mice in each group). **o**, Effects of Kaem and Rhap on the expression levels of different p-Tau sites (Thr181, Ser202/Thr205, Thr217 and Thr231) in hippocampal tissues from the 3xTg AD mice (*n* = 3 mice per group). All quantitative data are shown as mean ± s.e.m. Two-way ANOVA followed by Tukey’s multiple comparisons test (**b**–**g**, **i**–**l**, **n**). NS, no significance; *P < 0.05, **P < 0.01, ***P < 0.001. Additional data on the mechanisms of mitophagy induction by Kaem and Rhap in mice are shown in Extended Data Fig. 4. Original western blot gels for **o** are included in Source Data Fig. 3.

Encouraged by the in silico, in vitro and nematode data, we wondered whether the anti-AD effects would translate to rodents. Therefore, we decided to test this using classical 3xTg AD mice bearing both Aβ and Tau pathologies^54^. We treated the 3xTg AD mice from 12.5 months with both compounds (100 mg kg^−1^ d^−1^) via oral gavage for 2 consecutive months and subsequently assessed memory and pathologies. In line with the results obtained using the *C. elegans* AD models, both Kaem and Rhap greatly improved spatial learning and memory in the Morris water maze test in terms of latency to the platform from days 1 to 6 (Fig. 6a,b), and in-platform frequency for the probe trial at day 7 (Fig. 6c). Similarly, Kaem and Rhap improved spatial memory in 3xTg AD mice tested using the Y maze spontaneous alternation performance test (Fig. 6d). Both molecules enhanced visual recognition memory when tested with a novel object recognition (NOR) test (Fig. 6e) compared with WT mice (Veh).

**Extended Data Fig. 4:**
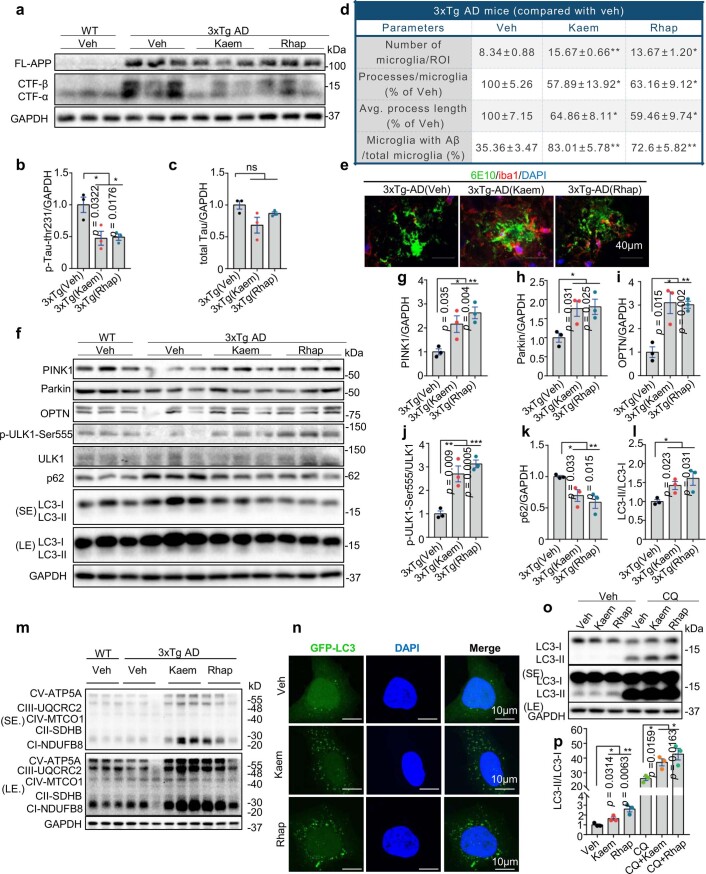
Kaem and Rhap inhibit Aβ production, increase microglial phagocytosis, and induce mitophagy and autophagy. **a**, Effects of Kaem and Rhap on protein levels of full-length APP (FL-APP), CTF-α and CTF-β in hippocampal tissues from 3xTg AD mice (n = 3 per group). **b**, **c**, Quantification of phosphorylated Tau sites (Thr231) and total Tau/GAPDH in hippocampal tissues from 3xTg AD mice (n = 3 biologically independent samples). **d**, **e**, Effects of Kaem and Rhap on microglial phagocytosis of Aβ plaques in hippocampal tissues from 3xTg AD mice. Data were quantified from 3 random images/mouse from a total of 3 mice (**d**). Aβ plaques are shown in green (6E10 antibody) and microglia (anti-Iba1 antibody) are in red (**e**). **f**, Western blot results showing the effects of Kaem and Rhap on the levels of proteins involved in mitophagy (PINK1, Parkin, OPTN, p-ULK1-Ser555 and ULK1) and substrates of autophagy (p62 and LC3-II/I) in the hippocampal tissues of the mice (n = 3 mice per group). **g**–**l**, Quantification of Western blot data in (**f**), n = 3 biologically independent samples. se: short-exposure; le: long-exposure. **m**, Western blot results showing the effects of Kaem and Rhap on the levels of proteins involved in OXPHOS in the hippocampal tissues of the mice (n = 3 mice per group). **n**, Effects of Kaem and Rhap on autophagy induction using a HeLa cell line stably expressing GFP-LC3 following Kaem (10 μM) or Rhap (10 μM) treatment for 12 h before imaging. **o**, **p**, Western blot data (o) with semi-quantification (p) showing the effects of Kaem and Rhap on levels of LC3-II/I in the HEK293 cells (n = 3 biologically independent samples). All quantitative data shown in mean ± S.E.M. One-way ANOVA followed by Šidák’s multiple comparisons test (**b**–**d**, **g**–**l**, **p**) was used for data analysis. NS, no significance, *p < 0.05, **p < 0.01, ***p < 0.001. Original unprocessed western blots for **a**, **f**, **m**, **o** are available in Source Data Figs. 2–4.

We further asked whether improved cognition in the 3xTg AD mice was due to the alleviation of Aβ and Tau pathologies. Indeed, Kaem and Rhap comparably reduced insoluble Aβ_1–40_ (Fig. 6f), as well as soluble and insoluble levels of Aβ_1–42_ (Fig. 6g) in the hippocampal tissue of the 3xTg AD mice; consistently, an anti-Aβ_1–16_ (clone 6E10)-antibody probed immune-fluorescent evaluation showed reduced Aβ load per region of interest (ROI) (Fig. 6h,i). Mechanistically, Kaem and Rhap reduced Aβ production as supported by decreased levels of FL-APP, CTF-β and CTF-α in the hippocampal tissue of the 3xTg AD mice (Fig. 6j–l and Extended Data Fig. 4a), mirroring the results seen in the N2a mouse neuroblastoma cells (Extended Data Fig. 1c–f). While microglia use energy (generated mainly by mitochondria) to clear Aβ plaques, we recently reported over 60% reduction of microglial mitophagy in the hippocampal tissue from an APP/PS1 mouse model, which could be a cause for the reduced phagocytosis of Aβ plaques in AD; mitophagy stimulation increased microglial phagocytosis in the APP/PS1 mice^11,55^. Accordingly, we evaluated whether Kaem and Rhap influenced microglial activity and whether the increased microglial activity could enhance the removal of Aβ plaques. Indeed, Kaem and Rhap increased phagocytic engulfment of Aβ plaques by microglia from 35.4% (microglia with Aβ/total microglia, Veh Group) to 83.0% and 72.6%, respectively (Extended Data Fig. 4d,e). Kaem and Rhap also increased microglial populations in the AD hippocampus from 8.3 (number per region of interest) to 15.7 and 13.7, respectively (Extended Data Fig. 4d). Further, Kaem- and Rhap-treated mice displayed a decreased number and length of microglial processes, highlighting their shift towards a phagocytic state (Extended Data Fig. 4d).

We then examined aggregation levels of Tau in response to Kaem and Rhap treatment. While Kaem reduced 62.2% of AT8 (for p-Tau Ser202/Thr205)-positive cells, Rhap abrogated 73.5% of AT8-positive cells in the hippocampus of the 3xTg AD mice (Fig. 6m,n). At the molecular level, Kaem and Rhap dramatically reduced the phosphorylation levels of multiple classical p-Tau sites, such as Thr181, Ser202/Thr205 and Thr231, by over 50% (Fig. 6o and Extended Data Figs. 1j,k and 4b,c). Recent clinical studies point to a new p-Tau site, p-Tau217, which increases during early AD and correlates with AD with high sensitivity and discriminative accuracy^56,57^. To this end, we evaluated the effect of Kaem and Rhap on p-Tau217. Intriguingly, Kaem and Rhap caused a 57.8% and a 66.6% reduction in p-Tau217 expression, respectively (Fig. 6o and Extended Data Fig. 1l). To investigate whether the Kaem- and Rhap-induced reduction in p-Tau was dependent on mitophagy, we knocked down *PINK1 gene* in HEK 293 (with Tau P301L) cells. Immunoblotting data show that *PINK1* knockdown almost completely annulled the effect of Kaem and Rhap on the phosphorylation status of Tau, suggesting that these mitophagy inducers inhibit p-Tau (thr181) and p-Tau (thr217) via a PINK1-dependent pathway (Extended Data Fig. 1m–o).

We further checked mitophagy status in the 3xTg AD mice. Immunoblotting data from the hippocampal tissue show that major mitophagy proteins, including PINK1, Parkin, OPTN and p-ULK1 (Ser555), were reduced in the 3xTg AD mice when compared with WT mice. Meanwhile, levels of these proteins were higher in Kaem- and Rhap-fed AD mice (Extended Data Fig. 4f–j). In line with our results from cells and worms, our mouse data suggest that Kaem and Rhap restore impaired mitophagy in the 3xTg AD mice. In line with the immunoblot data, electron microscopic analysis shows accumulated mitochondria in the hippocampal tissue of the 3xTg AD mice, while this phenomenon was alleviated with Kaem or Rhap treatment accompanying increased mitophagy (Fig. 3n and Extended Data Fig. 1b). Furthermore, Kaem and Rhap increased protein expression levels of members of the mitochondrial OXPHOS complex (CV-ATP5A, CIII-UQCRC2 and CI-NDUFB8) in hippocampal tissue of 3xTg AD mice (Extended Data Fig. 4m).

While basal autophagy was lower in the hippocampal tissue of the 3xTg AD mice (lower LC3B-II/LC3B-I and higher p62) compared with WT mice, administration of Kaem and Rhap increased autophagy in the 3xTg AD mice (Extended Data Fig. 4f,k,l). Indeed, Kaem and Rhap promoted autophagy in vitro at 10 µM (Extended Data Fig. 4n–p), a dose that also triggers mitophagy in vitro (Fig. 2c). However, Kaem- and Rhap-induced memory improvement in worms is dependent on key mitophagy genes, such as *pink-1*, *pdr-1* or *dct-1/NIX* (Figs. 4c(left) and 5e, and Extended Data Fig. 3c). In addition, Kaem- or Rhap-induced inhibition of multiple p-Tau sites (Thr181 and Thr217) was dependent on the mitophagy gene *PINK-1* (Extended Data Fig. 1m–o). Given the intertwined linkage between mitophagy and autophagy, we do not exclude the possibility of a Kaem- and Rhap-induced non-mitophagy-related autophagy in the contribution to the multiple behavioural and pathological benefits reported here. Collectively, these combined behavioural, histological and molecular data indicate the bioavailability and strong capacity of Kaem and Rhap to retard memory loss, while simultaneously decreasing both Aβ and Tau pathologies in the 3xTg AD mouse model.

## Discussion

**Extended Data Fig. 5:**
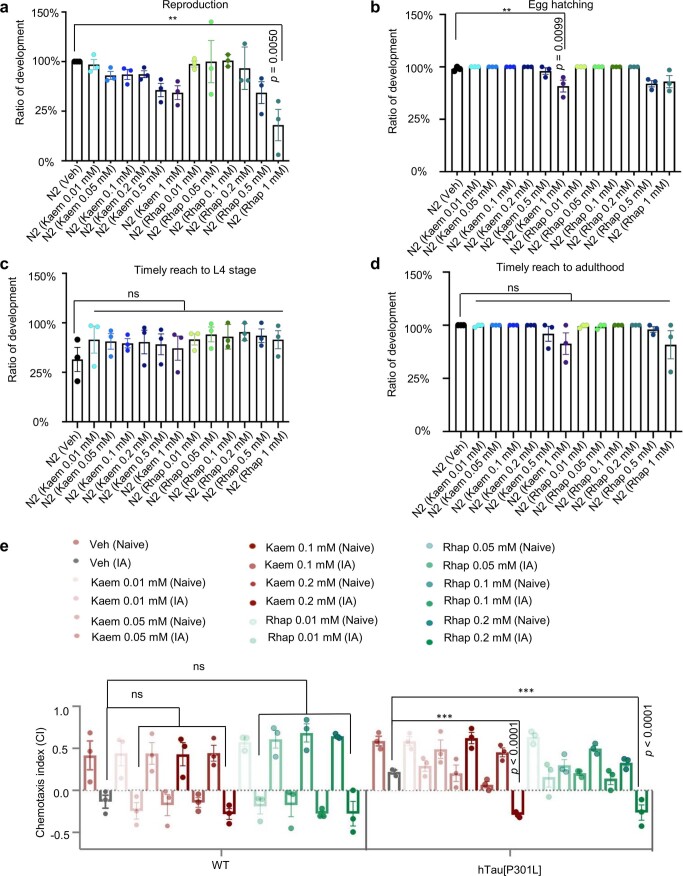
Kaem and Rhap are toxicologically safe to nematodes up to 1 mM and they do not show memory retention activity until reaching to 0.2 mM. Toxicological effects of Kaem or Rhap (0.01, 0.05, 0.1, 0.2 mM from egg hatching onwards) on fecundity (3-h egg-lay) (**a**), egg hatching (**b**), development to L4 (**c**), and time to reach adulthood (**d**). **e**, Effects of different doses of Kaem and Rhap (0.01, 0.05, 0.1, 0.2 mM from egg hatching onwards) on associative memory in transgenic animals expressing hTau[301 L]. All quantitative data are shown in mean ± S.E.M. from three biological repeats. Two-way ANOVA followed by Tukey’s multiple comparisons test was used for data analysis. NS, no significance, ***p < 0.001.

Aiming to unveil additional AD aetiologies and support the development of novel anti-AD drug candidates, we have established an in silico-in vitro-worm-mouse screening technology and validation approach to identify novel mitophagy modulators. Our method revealed two bioavailable neuronal mitophagy inducers with anti-AD potential, as they showed conserved memory retention capacity in one Aβ nematode model, two Tau nematode models and the 3xTg AD rodent model. Compared with most clinical-trial-stage anti-AD drug candidates, which target only Aβ or Tau pathology^7^, these mitophagy inducers work on both. Mechanistically, they inhibit Aβ pathology via production inhibition and microglial phagocytic clearance, abrogate Tau pathology via inhibition of multiple p-Tau sites (including p-Tau181, Ser202/Thr205, Thr231 and Thr217) and reduce spreading of pathological Tau. Kaem and Rhap are likely biological safe as the doses used for *C. elegans* and mice did not show detectable side effects (Extended Data Fig. 5 and Fig. 6). E.g., based on the toxicity assay results, treatments with Kaem or Rhap at concentrations ranging from 0.01 to 1 mM did not influence worm development (Extended Data Fig. 5a–d).

Over the past years, the scope of AI has moved from sheer theoretical knowledge to real-world applications, including drug development for aging and various diseases^17,19,22,58^. Our AI-driven virtual screening recommendation has achieved a 44% (8/18) success rate, which is much higher than traditional screening methods for small compounds. For instance, typical rates from experimental high-throughput screening have ranged between 0.01% to 0.5%, while typical hit rates for prospective virtual screening have been reported at 1%–10%^59,60^. Due to the low numbers of reference compounds, that is, mitophagy inducers (Supplementary Table 1), we have not been able to identify candidate compounds using machine learning approaches that may require large numbers of reference compounds (typically requiring between 100 to over 1,000 compounds)^19,22^. To overcome the shortage of reference compounds, we have chosen to use a combinational approach, combining simplified molecular-input line-entry system (SMILES) sequences, molecular interaction features and 3D conformers fingerprint, which can represent the molecule as a fixed bit-string on the basis of expert-encoded substructures, but do not require big training datasets^23^. However, since the fingerprint techniques rely heavily on the manually designed functional groups in the local chemical environment, they cannot capture global structural and pharmacophore information, and thus usually underperform in the context of complicated biological systems^61^. More recently, several fingerprint-inspired unsupervised machine learning approaches (for example, Mol2vec^26^ and N-gram^26^) have rapidly advanced to utilizing larger compound datasets such as ChEMBL and ZINC for the comprehensive representation of molecules^24,25^, yielding gains in property prediction performance over manually crafted fingerprints and preliminary machine learning methods. Compared to the sparse fingerprint vectors, our method has provided an efficient and information-enriched molecular embedding technique via learning the global relationship and ‘semantic similarity’ of each component. This has encouraged us to bypass the large dataset required by an unsupervised machine learning regime and has established a pipeline for virtual screening in complicated biological systems on the basis of only a few data points. Our machine learning approach could be applied to the screening of additional new mitophagy stimulators in larger drug libraries. Advanced AI technologies, such as a trained deep neural network^62^, could be used to predict mitophagy induction activity in molecules that are structurally different from known mitophagy inducers in the future, when larger numbers of reference compounds become available.

Our findings reveal a conserved mechanism for memory loss that is at least partially mediated by defective mitophagy, while our in silico, high-accuracy screening technology paves the way for identifying exploitable mitophagy stimulators to improve healthspan and cognition in humans. Since mitochondria are essential for neuronal plasticity and survival, we have postulated that defective mitophagy driven by genetics, aging and/or other environmental factors, displays a causative role ahead of Aβ and Tau pathologies in AD development and progression^1,2,11^. The current study further consolidates this hypothesis since key mitophagy pathways, including the PINK1/Parkin pathway, possess a central role in memory improvement upon supplementation with mitophagy-inducing compounds. Kaem is a natural flavanol available in a variety of plants including beans, tea, kale and broccoli^63^. Rhap is a stilbenoid/phytoalexin, an antimicrobial compound produced by plants, such as *Vitis* species (grapes), etc. A well-known natural analogue of Rhap is resveratrol, which shows anti-aging and neuroprotective activities in cross-species animal studies and clinical trials^64,65^. Preclinical studies have shown that the anti-AD potential of Kaem may lie in its anti-oxidative and anti-inflammatory activities^66,67^. Indeed, a recent ongoing community-based prospective cohort study with a total of 921 participants, suggests that taking flavonols, including Kaem, reduces the risk of AD^63^. The current study verifies the anti-AD potential of Kaem using both nematode and rodent models of AD, and shows that mitophagy induction is a major molecular mechanism of neuroprotection for AD. We also identified Rhap as a robust mitophagy inducer able to potentially inhibit Aβ and Tau pathologies in both worm and mouse models of AD, in a largely mitophagy-dependent manner. The pharmacokinetic data for both Kaem and Rhap are available. Kaem can cross the blood–brain barrier;^68–70^ whether Rhap is able to cross the blood–brain barrier has yet to be determined. Future clinical studies should address the appropriate dose range, delivery routes and any potential side effects. To note, our mitophagy-targeting drug screening strategy also reduces the risk of eliminating potential anti-AD drug candidates that may have little or no capacity to induce mitophagy. Among the 18 AI-selected molecules, Tacrolimus was able to induce mitophagy in HeLa cells but not in worm neurons. However, Tacrolimus was able to improve memory in the hTau(P301L) *C. elegans* model at a concentration of 0.2 mM (but not at 1 mM) (Supplementary Fig. 5b). Tacrolimus, a calcineurin inhibitor and an immunosuppressant, inhibited Aβ-induced dendritic spine loss^71^ and is scheduled for a pilot open-labelled clinical trial (NCT04263519).

Efficacy and safety determine clinical success in drug development^72^. A challenge in virtual screening and computer-aided drug design is the existence of ‘pan-assay interference compounds’ (PAINS), which are chemical compounds that normally cause false-positive results in high-throughput screens^73–76^. The application of carefully selected PAINS-related filters into machine learning/AI-based drug screening^75^, including the one we generated here, should be used. While we do not exclude the possibility that Rhap and Kaem could be hydroxylated, our strategy involving sophisticated in vitro analyses and multiple bioassays across two distinct in vivo experimental systems consistently shows the specificity and efficacy of the two lead compounds. Furthermore, the value of the effective dose of a drug candidate is a pivotal indicator of the potential of clinical studies. While the in vitro cell culture-based effective doses for many of the promising drug candidates are in the nM to 10 µM range^7,77^, there are drug candidates with effective doses in the mM range. For example, while nicotinamide adenine dinucleotide (oxidized form, NAD^+^) is reduced in aging and AD, there are many clinical trials on the application of NAD^+^ precursors, such as nicotinamide riboside and nicotinamide mononucleotide, in treating neurodegenerative diseases and multiple age-related diseases^5^. The effective doses for nicotinamide riboside/nicotinamide mononucleotide in cell culture are between 0.5 to 3.0 mM; the doses used in clinical trials range from 250 to 2,000 mg d^−1^ without detectable toxicity^5,78^. For Kaem and Rhap, no detectable toxicity was observed at 50 µM in cells (effective dose = 10 µM) (Extended Data Fig. 3l), at 0.5 mM in *C. elegans* (effective dose = 0.2 mM) (Extended Data Fig. 5a–d) or at (100 mg kg^−1^ d^−1^) in mice (Fig. 6). These data strongly indicate that Kaem and Rhap are effective and safe in both cells and animals.

It is intriguing that aging and defective mitophagy converge with AD-defining Aβ and Tau pathologies to inflict synaptic loss and neuronal death leading to subsequent cognitive dysfunction and brain homeostatic collapse. Although hyperactivation of mitophagy may not be without detrimental consequences for health, targeted mitophagic restoration is able to alleviate memory loss in a diverse set of nematode and rodent models of AD^11,15,79^. The broader impacts of mitophagy in targeting multiple aspects of AD pathogenesis, such as impaired proteostasis, inflammation, neuronal death, glial dysfunction and energy metabolism^1,11,80^, support the potential value of examining mitophagy inducers for AD mitigation in a clinical setting.

## Methods

### Machine learning-based screening of mitophagy inducers

Multi-representation models (1D, 2D and 3D) were first pre-trained to generate representations for compounds. The goal of the pre-training process is to generate comprehensive molecular representations for different molecules. The pre-training models can generate vector representations for molecules that are not included in the initial pre-training dataset. The compounds from the ChEMBL and ZINC database were filtered using the parameters noted in Jaeger et al^26^. We transformed the compounds into SMILES representations using RDKit^81^, which simplified multi-dimensional molecular structures into ordered text chains. In total, 19.9 million molecules were included in all further processes (hereafter called the pre-training dataset). A Mol2vec model was pre-trained on the basis of the pre-training dataset and new molecules could be described by summing the substructure vectors retrieved from a pre-trained Mol2vec model. The 2D pharmacophore fingerprint was implemented to introduce the fragment graph relationship between different molecules. Ehrlich^82^ defined a pharmacophore as “a molecular framework that carries (phoros) the essential features responsible for the biological activity of a drug (pharmakon)”. Therefore, the structural similarity between different molecules could be captured on the derived distance between the topological distributions of the various atoms that were inherent in the function of the molecules themselves. The 2D pharmacophore fingerprint was obtained using RDKit^81^, producing a vectorized prioritization of molecules based on the 2D topological similarity (that is, the fingerprint). The 3D conformers fingerprint method, initially described by Landrum et al., was introduced to augment the 3D information^83^. The original dataset (with an initial 19.9 million compounds) was again run through RDKit using the 3D conformer fingerprint module. This technique used information about the features (chemically active areas) on a molecule, producing a ‘feature-map vector’ that captured information around the projected biological activity of molecules.

We applied the 1D, 2D and 3D fingerprints to identify potential mitophagy inducers within the screening process. The similarity score for each of the 3,724 compounds with the filtered molecules was calculated, with a higher score indicating a higher degree of similarity. We defined the strength of the relationship from the three models trained by different information, that is, 1D—molecules semantic information, 2D—fragments graph relationship and 3D—atomic bonds, spatial angle and length as the similarity. The similarity score was calculated as:

1 $$s = v \ast w,$$

where *s* is the similarity score, * is the vector dot product, and *v* (the inductor vector) and *w* (the compound vector) are high-dimensional vector representations from the pre-trained model.

We clustered the representation based on the 14 mitophagy inducers scored by the similarity function (equation 1). We then filtered the outliers and ranked the low-score compounds on the basis of filter function (equation 2).

2 $${{{\mathrm{filter}}}}\;{{{\mathrm{function}}}} = \left\{ {\begin{array}{*{20}{c}} 0 & {s = 1} \\ s & {\theta \le s < 1} \\ 0 & {s < \theta } \end{array}} \right.,$$

where *s* is the similarity score and *θ* stands for the cut-off value (=0.75). The molecules with a similarity ranking score ≥0.75 were selected. In total, the selection method described here produced 18 novel analogue compounds for validation (Supplementary Table 2).

#### Small compounds in the Macau Library

All AI-selected compounds were from the State Key Laboratory of Quality Research in Chinese Medicine, Institute of Chinese Medical Sciences, University of Macau, Macau SAR, China (J.-H.L.). All molecules were of at least 98% purity and dissolved in dimethylsulfoxide (DMSO) for experiments. For the in vitro and in vivo experiments, Kaem (HY-14590) and Rhap (HY-N2229) were purchased from MedChemExpress, with 99.62% and 99.66% purity, respectively.

#### *C. elegans* strains and genetics

A list of strains used in this study is shown in Supplementary Table 6.

#### Drug treatment of *C. elegans*

All the *C. elegans* strains were maintained at 20 °C in standard nematode growth medium (NGM) plates with OP50 as a food resource^84^. All the experiments were performed at 20 °C, unless specified elsewhere. DMSO-dissolved compounds (Kaem, Rhap, Quercetin, Ascomycin, Tacrolimus, Isorhamnetin and Pinostilbene) were added just before pouring the NGM plates. Worms were treated with designated drugs from either egg hatching or L4 stage, or other time points as specified elsewhere.

### mRNA quantification using *C. elegans* tissue

Real-time PCR was performed as previously described^85^. Worms of designated age were collected, washed with M9 buffer, followed by isolation of total RNA using TRIzol (Thermo Fisher, Catalog no. 15596026). For complementary DNA synthesis, messenger RNA was reversely transcribed using an iScript cDNA Synthesis Kit (catalog no. 1708890, Bio-Rad) for 5 min at 25 °C, 20 min at 46 °C, 1 min at 95 °C and finally at 4 °C for storage. cDNA samples were then used for standard real-time quantitative reverse transcription PCR (real-time qRT-PCR) to quantify mRNA levels of *dct-1*, *pink-1*, *pdr-1*, *bec-1*, *sqst-1*, *vps-34*, *skn-1* and *rheb-1* mRNA using the following primers:

*dct-1:* 5′-GGCTCCAACCTTACCACTCC-3′ and 5′-GCAAATCCTACT GCTGCTCC-3′;

*pink-1:* 5-AGCATATCGAATCGCAAATGAGTTAG-3′ and 5′-TCGACCGTGGCGAGTTACAAG-3′;

*pdr-1:* 5′-AGCCACCGAGCGATTGATTGC-3′ and 5′-GTGGCATTTTGGGCATCTTCTTG-3′;

*bec-1:* 5′-CTGTCAGCATCCGTTGAGGT-3′ and 5′-AGAGCGTCAGAGCAATCATTACA-3′;

*sqst-1:* 5′-ATCCGCTCCTCACCAAATGC-3′ and 5′-TGTTGGACGAAGGGGAACAG-3′;

*vps-34:* 5′-ATGATTCCAGGTATGCGGGC-3′ and 5′-CTGACGAGCAAGTTGAGAGGA-3′;

*skn-1*: ACAGTGCTTCTCTTCGGTAGC-3′ and 5′-GAGACCCATTGGACGGTTGA-3′; and

*rheb-1*: 5′-ACAAGACGGATCTCAGCACG-3′ and 5′-TCGAACACCTCATGCACTCG-3′.

Quantitative PCR was performed in triplicates, and the real-time PCR reactions were performed using QuantStudio 7 Flex System v1.1 (Applied Biosystems by Life Technologies) by heating to 95 °C for 10 min, cycling 40 times between 95 °C for 15 s, 60 °C for 1 min, and taking a melt-curve analysis between 95 °C for 15 s and 60 °C for 1 min.

### Evaluation of neuronal mitophagy inducers using a *C. elegans* mitophagy reporter strain

A *C. elegans* neuronal mitophagy reporter strain (neuronal mt-Rosella) was used to quantify neuronal mitophagy in worms as previously described:^11^ transgenic animals expressing a pan-neuronal mt-Rosella biosensor that combines a GFP variant sensitive to the acidic environment of the lysosomal lumen, fused to the pH-insensitive *Discosoma* red fluorescent protein (DsRed). Mitophagy was calculated as GFP/DsRed. Thus, the lower the ratio of pixel intensity, the higher the level of mitophagy. For all nematode experiments, compounds (0.01–1 mM) were administered from egg stage onwards. Unless specified, adult day 1 (the day after the L4 stage) nematodes were used for the experiments. Moreover, transgenic nematodes expressing the DCT-1::GFP mitophagy reporter, together with the autophagosomal marker LGG-1::DsRed in neurons were used to quantify mitophagy as previously reported^11^. Kaem and Rhap (0.01–1 mM) were administered from egg hatching onwards. Images of adult day 1 worms were taken using confocal microscopy, with co-localization between LGG-1 and DCT-1 serving to quantify the number of mitophagy events.

### *C. elegans* short-term memory assay

Chemotaxis to soluble and volatile compounds (isoamyl alcohol, IA) was performed at 20 °C on 10 cm agar plates as previously described^11,86^. In step 1, synchronized worms (around 200 worms per group) were collected and washed 5 times with M9 buffer, followed by placement in plain NGM plates (with no OP50) with/without IA for 90 min. In this step, for the IA conditioning plate, a droplet of 10 μl pure IA was placed on the middle of the lid. In step 2, to prepare assay plates, 20 µl 20 mM NaN_3_ was added on ‘IA’ and trap ‘T’ points, respectively. Assay plates were left at room temperature (20–22 °C) for 30 min to dry before testing. A 0.5 × 0.5 cm Parafilm was placed over the ‘IA’ area of each plate. In step 3, worms from step 1 were collected via washing with M9 buffer, followed by placing the rinsed worms on the source point ‘S’ area (additional M9 buffer was quickly dried with tissue paper). Four μl diluted IA (1/50) was added on the Parafilm placed on the ‘IA’ area. The worms were left at room temperature for 2 h. For step 4, the number of worms in different regions were counted, including the ‘S’, ‘IA’ and ‘T’ areas. The chemotaxis index was calculated as (#‘IA’ − #‘T’)/(#‘IA’ + #‘T’ + #‘S’), where ‘#’ denotes numbers^87^. A smaller score correlates with better memory performance. Three to six biological replications were performed for all nematode experiments. The memory assay was performed using adult day 1 worms, unless specified elsewhere.

### *C. elegans* lifespan assay, pharyngeal pumping rate, mobility and toxicity assays

Lifespan analysis was performed at 20 °C as previously described^88^, using 3.5 cm NGM plates with *E. coli* OP50 seeded 3 d ahead of experiments. Drugs (final concentrations were 0.2 mM Rhap, and 0.2 mM Kaem) were added during pouring of the NGM plates. Synchronous animal populations were generated via a 6 h egg lay by gravid adults to obtain tightly synchronized embryos that were allowed to develop into adulthood under defined conditions. Around 35–40 L4 stage worms (day 0) were transferred to 3.5 cm plates to obtain synchronous populations of at least 100 animals per condition. Animals were scored as dead or alive and transferred every 2–3 d to fresh plates seeded with OP50 during the fertile period, and then every 5 d until death. Worms were examined every day and were considered dead when they had stopped pharyngeal pumping and were unresponsive to touch. Worms that died due to internal bagging, crawling on the edge of the plates or gonad extrusion were scored as censored. These animals were included in lifespan analyses up to the point of censorship and were weighted by ‘0’ in mortality calculations. Parameters like mean, standard deviation of the mean and *P* value were calculated using the log-rank test (Mantel–Cox) from a pooled population of animals. Kaplan–Meier (K–M) survival curves were used for lifespan presentation. We used the GraphPad Prism software package for statistical analysis and to determine lifespan values. For evaluations of pharyngeal pumping rates, worms were synchronized and raised to adults as mentioned in the lifespan assay methodology. At designated ages, pharynx contractions were manually counted for 30 s^88^. *C. elegans* movement analysis was performed as described^89^. Briefly, worms were exposed to designated concentrations of Kaem (0. 2 mM) and Rhap (0. 2 mM) beginning at egg stage and the maximum velocity of movement was determined at adult day 1. Fifty to 100 nematodes were placed in one plate and recorded for 1 h and 30 min using the Movement Tracker system (Nemamtrix) to assess mobility. Three biological repeats were carried out. A double-blinded approach was used to ensure objectivity. Toxicity experiments, including fecundity (3 h egg-lay), egg hatching and larval development, were conducted using N2 *C. elegans* at 20 °C as detailed elsewhere^90^. Briefly, synchronized eggs were placed on NGM plates seeded with *E. coli* (OP50). L4 larvae were subsequently transferred onto fresh OP50-seeded NGM plates and allowed to grow to adulthood. Ten adult day 1 worms were transferred onto assay NGM plates with OP50 and containing either Kaem (0.01, 0.05, 0.1, 0.2, 1 mM), Rhap (0.01, 0.05, 0.1, 0.2, 1 mM) or vehicle control. Three plates of worms for each group were set up to achieve an *n* = 30 per experimental condition. The gravid worms were subjected to a 3 h egg-lay followed by removal of worms from the plates. The number of eggs laid was quantified as a measure of the reproductive capacity of the worms. The following day, the number of unhatched eggs and L1 larvae were counted to determine the efficiency of egg hatching. Development to L4 larvae was assessed 36 h later after egg laying as a measure of larval growth. Finally, the growth of L4 larvae to adulthood was quantified 16 h after the L4 larval stage.

### Evaluation of neuronal activity and neurodegeneration in *C. elegans*

Glutamatergic neurodegeneration was analysed using a well-established method reported elsewhere^40,91^. In brief, adult day 3 animals were prepared for confocal imaging. While there are around 15 glutamatergic neurons in the worm tail region^92^, 5 were the focus of this analysis: LUA (R), LUA (L), PVR, PLM (R) and PLM (L). An animal was scored as normal if all 5 neurons were present and without malformations such as axon breaks, swelling, distension or separation of the soma. In addition, GFP intensity of the PVR neurons was quantified using the software ImageJ-1.50, with data from 30 worms randomly selected in each group from 2 biological replicates. Only PVR neurons with no clear malformations such as axon breaks, swelling, distension or abnormal location in the tail, were used for intensity quantification. The experiments were repeated 2 times with 40–50 worms per group per experiment.

Cholinergic synaptic transmission assay was performed as previously described^43^. This assay evaluates the sensitivity of worms to the synaptic transmission of acetylcholine at the neuromuscular junction via monitoring the paralyzing effect of an acetylcholinesterase inhibitor, aldicarb. In the presence of aldicarb, breakdown of acetylcholine is inhibited, resulting in the accumulation of acetylcholine in the synaptic cleft. This build-up of acetylcholine results in over-activation of cholinergic receptors, followed by muscle hyper-contraction, paralysis and finally death. Briefly, synchronized adult day 1 worms, achieved via a 5 h egg-lay, were incubated in the presence of aldicarb at different concentrations and scored every 30 min for the time course of the aldicarb-induced paralysis. While a series of doses were used (vehicle, 0.25, 0.5 and 1 mM) for optimization, 0.5 mM was chosen for further rescue experiments (±0.2 mM Kaem or 0.2 mM Rhap, or as described elsewhere). Thirty worms per group were used for the experiments, with finalized data from 2–4 biological repeats. VC223 (a strain hypersensitive to aldicarb-induced paralysis) and NM204 (a strain resistant to aldicarb-induced paralysis) were used as controls. Data were analysed and presented using Prism 8.0 (GraphPad Software).

### Detection of in vitro mitophagy using mt-Keima fluorescent reporter

Imaging of mt-Keima HeLa cells was performed as reported elsewhere^31^, using different settings for GFP and red fluorescent protein (RFP). mt-Keima is a ratiometric pH-sensitive fluorescent protein that exhibits green fluorescence (excitation 458 nm) in basic or neutral conditions and red fluorescence (excitation 561 nm) in acidic conditions^33^. For our experiments, the settings used were green channel (excitation 458 nm, emission 570–695 nm) to visualize normal mitochondria, and red channel (excitation 561 nm, emission 570–695 nm) to visualize mitochondria undergoing mitophagy. The mt-Keima HeLa cells were treated with different drugs of designated doses for 24 h, followed by confocal imaging. A total of 5 randomly selected regions per sample was chosen for imaging, with a total of 3 biological repeats. Data were quantified, using ImageJ software, as the total number of red pixels divided by the total number of all pixels.

### Evaluation of Tau aggregation and degradation in HEK293 cells expressed with P301S Tau-Venus

HEK293 cells stably expressing the 0N4R isoform of human Tau, bearing P301S mutation with a C-terminal Venus tag treated with exogenous recombinant heparin-assembled P301S Tau (tau seeds) were used for the experiments. Exogenous Tau seeds promote the conversion of endogenous Tau Venus from a dispersed distribution to bright foci^49^. Both the HEK293 P301S Tau-Venus cell line and the Tau seeds were a gift from the Dr William A. McEwan Lab at the University of Cambridge. The HEK293 P301S Tau-Venus cells were cultured in DMEM (with 10% fetal bovine serum (FBS) and 1% penicillin–streptomycin (P&S)) in a humidified incubator at 37 °C with 5% CO_2_. On the day of the experiment, cells were trypsinized and seeded in Opti-MEM (Gibco, no FBS, no antibiotics) at a density of 5,000 cells per well per 50 µl in a 96-well plate (precoated with Poly d-lysine/PDL) and incubated for 24 h. To induce endogenous Tau aggregation, 50 μl Tau-containing Opti-MEM lacking FBS and antibiotics (20 nM Tau + 1% Lipofectamine 2000) was pre-incubated at room temperature for 10 min, then added to each well for 1.5 h incubation at 37 °C. Two types of experiments were performed: one to evaluate Tau aggregation and the other to quantify degradation of aggregated Tau. Experiment one was to evaluate how drugs affected seeded Tau-induced endogenous Tau aggregation: 50 μl DMEM (with 20% FBS and 1% P&S) containing vehicle (DMSO), Kaem (3.125, 6.25, 12.5, 25 μM) or Rhap (3.125, 6.25, 12.5, 25 μM) were added to respective wells, then incubated for 72 h. Cells were then fixed with 4% formaldehyde solution in 1× PBS for 10 min at room temperature. Cells were then stained with DAPI (1 µg ml^−1^) in 1× PBS for 10 min before imaging. For experiment two, to evaluate how drugs affect degradation of aggregated Tau, 50 μl DMEM (with 20% FBS and 1% P&S) was added, followed by 48 h incubation. The medium was replaced with 150 μl DMEM (with 10% FBS and 1% P&S) and vehicle (DMSO), or Kaem (3.125, 6.25, 12.5, 25 μM) or Rhap (3.125, 6.25, 12.5, 25 μM) for 24 h. Cells were fixed with 4% formaldehyde solution in PBS for 10 min at room temperature. Cells were stained with DAPI (1 µg ml^−1^) in PBS for 10 min before imaging. A fluorescent microscope (ZEISS Axio Zoom.V16) was used for imaging. One image per well was taken, with a total of 8 technical repeats per biological repeat. Data were analysed using ImageJ, from images from 3 biological repeats.

### Cell culture for mechanistic studies

The N2a, YFP-Parkin HeLa, Mito-GFP/mCherry-Parkin Hela, HEK 293 and GFP-LC3 HeLa cells were cultured in DMEM containing 10% FBS and 1% P&S. N2S (N2a cells expressing human Swedish mutant APP695) and HEK 293 cells expressing pTRE3G-mcherry-BI promoter-EGFP Tau P301L were maintained in DMEM supplemented with 10% FBS, 1% P&S and 200 μg ml^−1^ G418. A high concentration (approximately 1,000 μg ml^−1^) G418 was used for selection, and a low concentration (200 μg ml^−1^) was used for maintenance. The expression of EGFP-Tau P301L is controlled by the addition of doxycycline (DOX) to the culture medium before indicated treatments. All the cell lines were maintained in the incubator at 37 °C with 5% CO_2_. After designated treatments, cells were subjected to imaging (autophagy) or collected for western blot analysis. For the autophagy imaging, GFP-LC3 HeLa cells were fixed and stained with DAPI. The images were acquired using a confocal microscope (TCS SP8, Leica).

### Transgenic mice, behavioural, pathological and mechanistic studies

Mice homozygous for all three mutant alleles (3xTg AD; homozygous for the Psen1 mutation and homozygous for the co-injected APPSwe and tauP301L transgenes (Tg(APPSwe,tauP301L)1Lfa)) were obtained from the Jackson Laboratory. The 3xTg AD mice and age-matched C57 mice were housed in individually ventilated cages on standardized rodent bedding. Mice were housed under constant light cycle (12 h light/dark) with free food and water available. The 12.5-month-old 3×Tg AD mice were treated with Kaem or Rhap (100 mg kg^−1^ d^−1^) by oral gavage for 2 months, and subsequently evaluated for behavioural and molecular endpoints. All animal care and experimental procedures were approved by the Committee on the Ethics of Animal Experiments of the University of Macau (UMARE-013–2019).

### Mouse behavioural studies

Several behavioural assays, including Morris Water Maze (MWM), Y maze and NOR were used to investigate changes in learning and memory. The MWM test was performed as described previously^93^. The device is a circular white pool (120 cm diameter × 50 cm depth) filled with water dyed white with TiO2, and with temperature maintained at 22 °C. A 10-cm-diameter platform was placed 1 cm below the water surface at a fixed position. Mice were trained with 4 trials per day for 6 consecutive days. Each trial lasted 60 s or until the mouse found the platform. If the mouse did not find the platform during the allocated time period, the experimenter directed the mouse to the platform. After each trial, the mouse was placed on the platform for 10 s. On the 7th day, the platform was removed for a probe trial (60 s) to assess long-term spatial memory retrieval. All parameters were recorded by a video tracking system (Labmaze V3.0, Zhongshi Technology). The Y maze spontaneous alternation performance (SAP) test measures the ability to recognize a previously explored environment^94^. The maze consists of 3 arms (30 cm length × 20 cm height × 6 cm width) with an angle of 120 ° between each arm. Mice were introduced to the centre of the Y maze and allowed to freely explore the maze for 10 min. The maze was cleaned with 75% ethanol solution between animals to eliminate odour traces. The number of entries into each arm was recorded with Labmaze video tracking system. SAP is the number of subsequent forays into a novel arm over the course of 3 entries. The spontaneous alternation (%) = number of actual alternations/(total arm entries − 2) × 100. The NOR tests were performed as described previously^95^. The device is a grey plastic box (35 cm length × 35 cm width × 25 cm height). Mice could explore two identical objects for 5 min during the training phase; 2 h later, each mouse was returned to the box, which had been modified to contain one familiar object and one novel object. The box was cleaned with 75% ethanol solution between animals to eliminate odour traces. The Labmaze video tracking system was used to monitor exploration behaviour. Exploration time was calculated as the length of time each mouse sniffed or pointed its nose or paws at the object. The ‘recognition index’ refers to the time spent exploring the novel object relative to the time spent exploring both objects. For the behavioural studies, 7 mice were used. No statistical methods were used to predetermine sample sizes, but our sample sizes are similar to those reported elsewhere^96^.

### EM

The ultrastructure of mitochondria and mitophagosomes was visualized and imaged with EM using the method reported elsewhere^11^. In brief, after killing the mice with the standard approach and a quick collection of the designated brain tissue, Veh-, Kaem- and Rhap-treated mouse hippocampal tissues were fixed in 2.5% glutaraldehyde (pH 7.4) for 2 h. After three washes with 0.1 M phosphate buffer (pH 7.2), the tissues were fixed in 1% osmic acid at 4 °C for 2 h. The samples were gradient dehydrated in a series of ethanol baths. Subsequently, the samples were embedded in Epon-Araldite resin for penetration and placed in a mold for polymerization. After the semi-thin section was used for positioning, the ultra-thin section was made and collected for microstructure analysis, followed by counter-staining with 3% uranyl acetate and 2.7% lead citrate. The samples were then observed with an HT7800 transmission electron microscope. Three mice per group were used for EM examination, with 10–20 images randomly taken. Data were analysed manually in a double-blinded manner.

### Immunohistochemistry/Immunofluorescence

After the behavioural tests, anaesthetized mice were perfused with pre-cooled 1× PBS. Half brains were placed in 4% paraformaldehyde in PBS for 24 h and then equilibrated in 30% sucrose for 72 h at 4 °C; 1:8 series equidistant floating 30 μm coronal sections (240 μm interval) were prepared, including the dentate gyrus area. Approximately 9–10 slices from each mouse were incubated in blocking buffer (5% goat serum and 0.3% Triton X-100 in PBS) for 30 min at room temperature. Samples were then incubated overnight with the primary antibody at 4 °C and then incubated with the appropriate fluorescent probe-conjugated secondary antibodies for 1 h at room temperature while protected from light. Nuclei were stained with DAPI at a 1:1,000 dilution. Specific primary antibodies used included mouse anti-β-amyloid, 1–16 antibody (clone 6E10, catalog no. 803002, BioLegend), rabbit anti-iba1 antibody (019–19741, Wako) and mouse anti-AT8 antibody (cat no. MN1020, ThermoFisher). The slices were mounted with FluorSave reagent and visualized using the Leica TCS SP8 confocal microscope. Aβ plaques and AT8-positive cells per ROI were counted and quantified using ImageJ software. To assess morphological changes of microglial cells, ImageJ was used to measure numbers and length of processes per microglial cell^11,96^. To evaluate phagocytosis, numbers of iba1-positive microglia cells that were also Aβ-positive in the CA1 region were counted.

For the co-labelling analysis, rabbit anti-iba1 antibody (019–19741, FUJIFILM Wako Pure Chemical) and mouse anti-β-amyloid, 1–16 antibody (no. 803002, BioLegend) were used. Primary antibodies were visualized with Alexa-Fluor 488 and Alex-Fluor 555 secondary antibodies (ThermoFisher), and the cell nuclei visualized with DAPI. For the quantification of Aβ plaques, deposits larger than 10 μm were included. At least 3 random images per mouse from a total of 3 mice were used for quantification. To detect the co-localization of mitochondria and lysosomes after Kaem and Rhap treatments, LAMP1 antibody (no. sc-18821, Santa Cruz) was used to label lysosomes in Mito-GFP/mCherry-Parkin HeLa cells. A Leica TCS SP8 confocal microscope was used to capture pictures and ImageJ software was used to quantify the co-localization events. To detect the effects of the top 5 potential compounds on mitophagy induction, HeLa cells expressing mt-Keima were treated with designated compounds for 24 h, then imaged and quantified with a Zeiss LSM 880 Confocal System.

### Enzyme-linked immunosorbent assay (ELISA) for Aβ_1–40_ and Aβ_1–42_

ELISA assays were performed as reported previously, with minor modifications^97^. Mouse hippocampal tissues were homogenized individually with Qiagen TissueLyser-II homogenizer in radioimmunoprecipitation assay (RIPA) buffer (R0728, Sigma) containing protease and phosphatase inhibitors. Extracts were sonicated and spun at 100,000 *g* for 20 min at 4 °C. Insoluble pellets were extracted in 70% formic acid by sonication and spun at 100,000 *g* for 20 min. Samples were neutralized in 1 M Tris buffer (pH 11, 1:12 ratio dilution) and diluted in blocking buffer before loading on ELISA. Aβ in the RIPA fraction was regarded as the detergent-soluble fraction, while Aβ in the neutralized formic acid fraction was considered as the detergent-insoluble fraction. The ELISA plates were coated overnight with 6E10 (4 µg ml^−1^) antibody in coating buffer 0.1 M Na_2_CO_3_ (pH 9.6) and blocked with 4% Block Ace in PBS for 2 h. Equal amounts of the fractions were loaded in duplicate wells and incubated at room temperature for 2 h under shaking. Biotinylated 5C3 (no. 0060S, Nanotools) and 8G7 (no. 0061S, Nanotools) were used to determine Aβ_1–40_ and Aβ_1–42_, respectively. Secondary antibodies were diluted at 1:1,000 concentration in 1% Block Ace solution and incubated at room temperature for 2 h. After washing the plates with PBS with 0.1% Tween20 (PBST), streptavidin-HRP was added and the plates were incubated at 37 °C for 1 h. TMB substrate (no. 555214, BD Biosciences) was then added to the plates, and plates were incubated at room temperature for 30 min. Finally, an equal volume of stop solution was added, and absorbance was measured at 450 nm.

### Western blots using materials from cells and mouse brain

Western blot assay was done as previously described^88^. Samples from different cell lines and mouse brain tissues were collected and prepared using 1× RIPA buffer containing protease and phosphatase inhibitors, and the protein concentrations tested using the bovine serum albumin (BSA) method. Proteins were separated on a NuPAGE 4–12% Bis-Tris Protein Gel at 200 V running for 1 h (catalogue no. NP0336BOX, Thermo Fisher Scientific) and set transfer system at 250 mA for ~2–3 h (for all the proteins from 15 kDa to 350 kDa) on PVDF membrane and then probed with various antibodies. Chemiluminescence detection was performed using a ChemiDoc XRS System (Bio-Rad Laboratories). Gamma adjustment was used to reduce the dark background when necessary. Quantification was performed using ImageJ. Antibodies used were as follows (all from Cell Signaling Technology, unless otherwise stated): PINK1 antibody (catalog no. ab75487, Abcam; no. A7131, ABclonal), Parkin antibody (no. NB100–91921, Novus), FUNDC1 antibody (no. ab74834, Abcam), LC3B antibody (no. NB100–2220, Novus), Beclin1 antibody (no. 3495s), phospho-DRP1 antibody (no. S616), DRP1 antibody (no. 8570s), p62 antibody (no. 8025s), MFN2 antibody (no. 94823s), phospho-ULK1 antibody (no. 5869s), ULK1 antibody (no. 6439s), AMBRA1 antibody (no. 24907s), OPTN antibody (no. A1845, ABclonal), Tau antibody (no. 46687s), p-Tau-thr181 (no. 12285s), p-Tau-thr231 (no. ab151559, Abcam), p-Tau-ser202/thr205 (no. MN1020, ThermoFisher), p-Tau-thr217 (no. 44–744, ThermoFisher), beta amyloid polyclonal antibody (no. 51–2700, ThermoFisher), Parkin Rabbit pAb (no. A0968, ABclonal), Tim23 antibody (no. 611222, BD Biosciences), Total OXPHOS Rodent WB Antibody Cocktail (no. ab110413, Abcam) and β-actin antibody (no. A5441, Sigma). Secondary antibodies included anti-mouse immunoglobulin G (IgG; catalogue no. 7076s) and anti-rabbit IgG (no. 7074s). Primary antibodies were used at a 1:1,000 dilution, with secondary antibodies used at a 1:2,000–5,000 dilution.

### Statistical analysis

Data are presented as mean ± s.e.m., unless otherwise specified. Two-tailed unpaired *t*-tests were used for comparisons between two groups. Group differences were analysed with one-way analysis of variance (ANOVA) followed by Šidák’s multiple comparisons test or two-way ANOVA followed by Tukey’s multiple comparisons test for multiple groups. Prism 8.0 (GraphPad Software) was used for the statistical analysis. *P* values <0.05 were considered statistically significant.

### Reporting Summary

Further information on research design is available in the Nature Research Reporting Summary linked to this article.

## Supplementary information

Supplementary Information Supplementary figures and tables.

Reporting Summary

Supplementary Data Analysis of chemical similarities of the top 18 AI-suggested compounds.

Supplementary Video Representative 3D image, showing the five designated tail neurons targeted for the assay: LUA (R), LUA (L), PVR, PLM (R) and PLM (L) neurons.

## Data Availability

The main data supporting the findings of this study are available within the Article and its Supplementary Information. The raw wet-lab data generated in this study are available from the corresponding authors on reasonable request. Restrictions apply to the AI-related data: all requests for raw and processed data will be reviewed by Mindrank AI to verify whether the request is subject to any intellectual property or confidentiality constraints. Source data are provided with this paper.

## Source data

Source Data Fig. 1 Unprocessed western blots for Fig. 2a.
Source Data Fig. 2 Unprocessed western blots corresponding to Fig. 5f and Extended Data Figs. 1c,m and 4o.
Source Data Fig. 3 Unprocessed western blots corresponding to Fig. 6o and Extended Data Fig. 4a,f.
Source Data Fig. 4 Unprocessed western blots corresponding to Fig. 3a,e and Extended Data Fig. 4m.

## Code availability

Custom code was specific to our computing infrastructure and mainly used for data input/output and parallelization across computers and graphics processors. The code, currently under a patent-examination process, is available from the corresponding authors on reasonable request.
