# Supplementary material for: Amelioration of Alzheimer’s disease pathology by mitophagy inducers identified via machine learning and a cross-species workflow
Source: Nat Biomed Eng. 2022 Jan 6;6(1):76–93. doi: 10.1038/s41551-021-00819-5 (PMC8782726; doi:10.1038/s41551-021-00819-5)
Supplement: Supplementary file 1 — Supplementary figures and tables. [file 41551_2021_819_MOESM1_ESM.pdf]

nature portfolio

https://doi.org/10.1038/s41551-021-00819-5

Supplementary information

Amelioration of Alzheimer's disease pathology by mitophagy inducers identified via machine learning and a cross-species workflow

In the format provided by the authors and unedited

Contents

Supplementary Fig. 1 l Chemical structures of the AI top-scored compounds.
Supplementary Fig. 2 l An *in vitro* HeLa-mt-Keima cell-based screening system identify mitophagy inducers as well as compounds which do not induce mitophagy.
Supplementary Fig. 3 l Top 5 compounds selected by the 1D- or the 3D-AI model do not induce mitophagy in HeLa cells.
Supplementary Fig. 4 l Detection of *in vivo* mitophagy using mt-Rosella.
Supplementary Fig. 5 l Effects of different compounds on associative memory in the hTau[P301L] worms and on glutamatergic neuroprotection.
Supplementary Fig. 6 l Effects of Kaem or Rhap on the acetylcholinesterase inhibitor aldicarb-induced paralysis in both Aβ and Tau worms.

Supplementary Table 1 l List of the known mitophagy inducers as reference
Supplementary Table 2 l Detailed information of the selected small compounds
Supplementary Table 3 l The hit rates for experimental HTS, computer aided, QSAR, and machine learning approaches.
Supplementary Table 4 l A summary of lifespan values under different conditions
Supplementary Table 5 l Quantified data showing the effects of Kaem or Rhap on the acetylcholinesterase inhibitor aldicarb-induced paralysis in both Aβ and Tau worms.
Supplementary Table 6 l List of worm strains used

Additional Supplementary information
Supplementary Dataset l Analysis of chemical similarities of the top 18 AI-suggested compounds (Microsoft Excel file).
Supplementary Video 1 l One representative 3-D image showing the five designated tail neurons targeted for the assay. They were LUA (R), LUA (L), PVR, PLM (R), and PLM (L) neurons. Also see Supp. Fig.5.

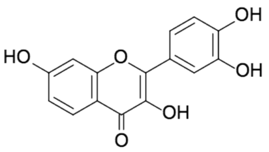

1. T2174

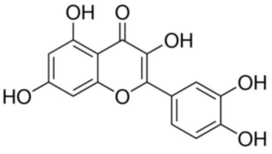

2. T6630

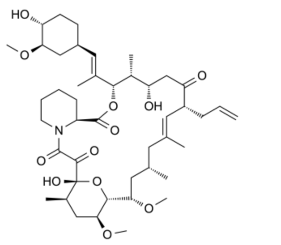

3. T2144

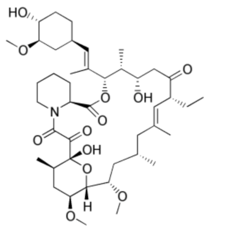

4. T2481

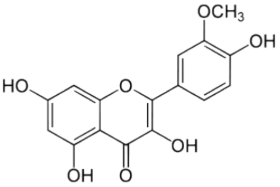

5. T2836

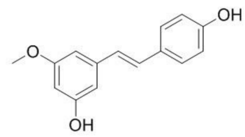

6. T3755

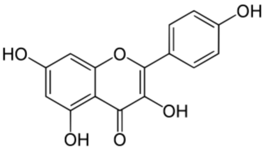

7. T2177

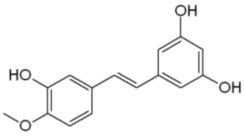

8. T3776

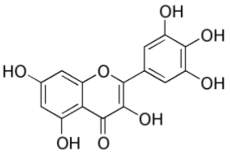

9. T0579

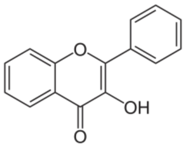

10. T2910

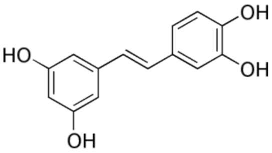

11. T0610

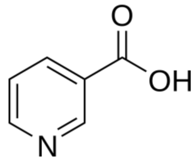

12. T0879

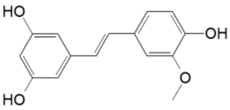

13. T3814

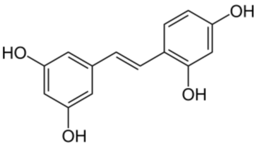

14. T3S1068

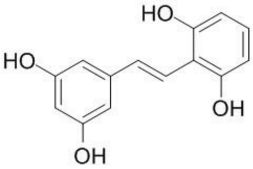

15. T7052

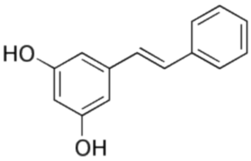

16. T3843

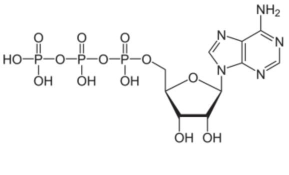

17. T2812

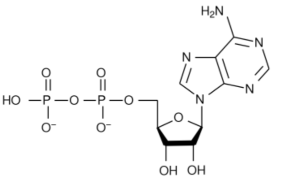

18. T1723

Supplementary Fig. 1 | Chemical structures of the AI top-scored compounds. Library IDs of each molecule are presented. For details, including names, CAS, molecular weight, etc., see Supp. Table 2.

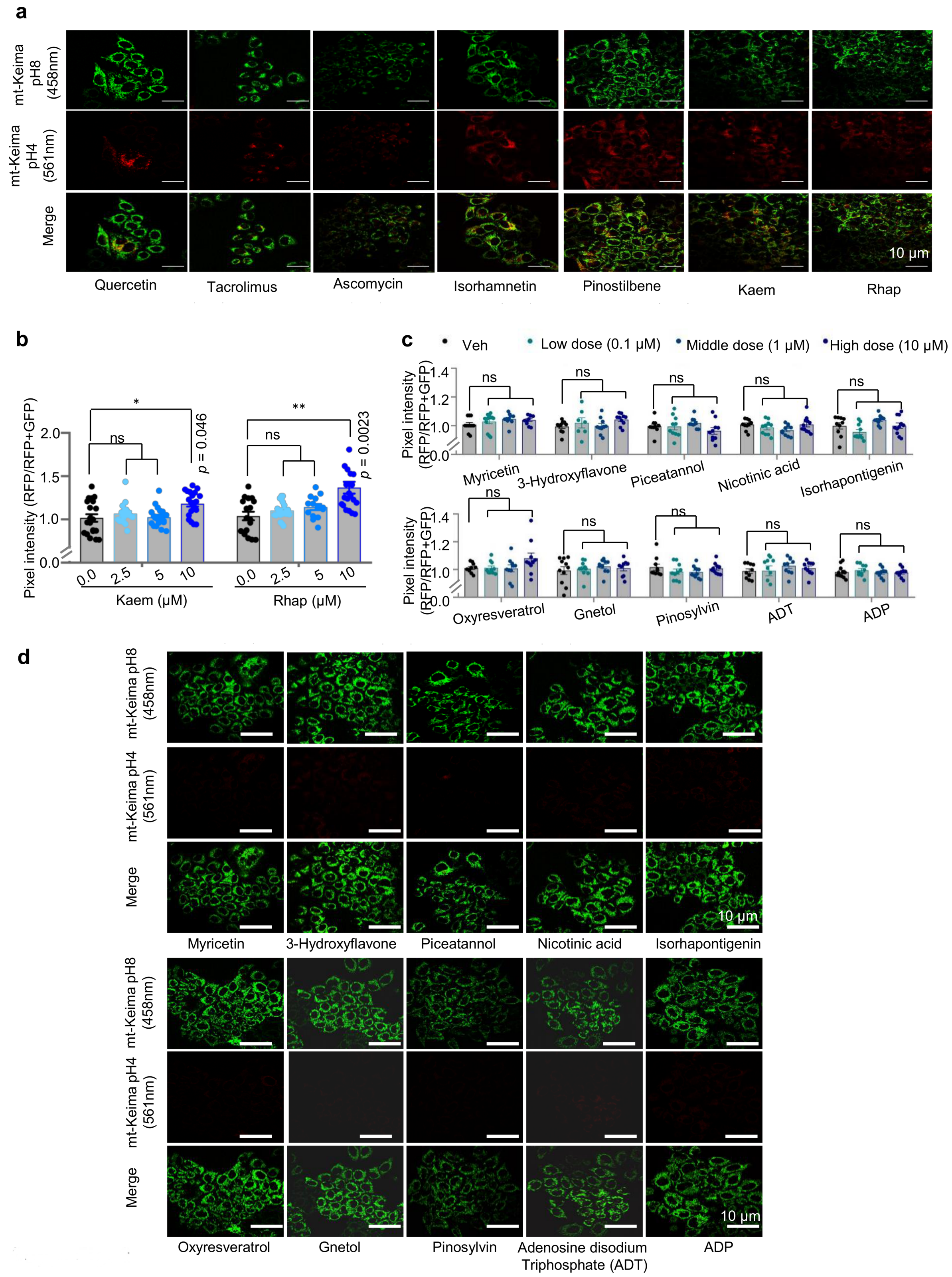

Supplementary Fig. 2 | An *in vitro* HeLa-mt-Keima cell-based screening system identify mitophagy inducers as well as compounds which do not induce mitphagy. a, Representative confocal images of quantitative data in Fig. 2b, 2c. HeLa-mt-Keima cells were treated with Quercetin, Tacrolimus, Ascomycin, Isorhamnetin, Pinostibene, Kaem and Rhap, respectively, at 10 *μ*M for 24 h before imaging via confocal microscopy. Scale bar, 10 µm. **b,** Effects of Kaem and Rhap (2.5 µM, 5 µM and 10 µM, 24 h) on mitophagy induction. **c,** Effects of designated compounds (from 0.1 µM to 10 µM, 24 h) on mitophagy induction in HeLa-mt-Keima cells, quantified data from two biological replicates. Two-way ANOVA followed by Tukey's multiple comparisons test was used for data analysis. **d,** One representative set of images of quantitative data shown in c. Scale bar, 10 µm.

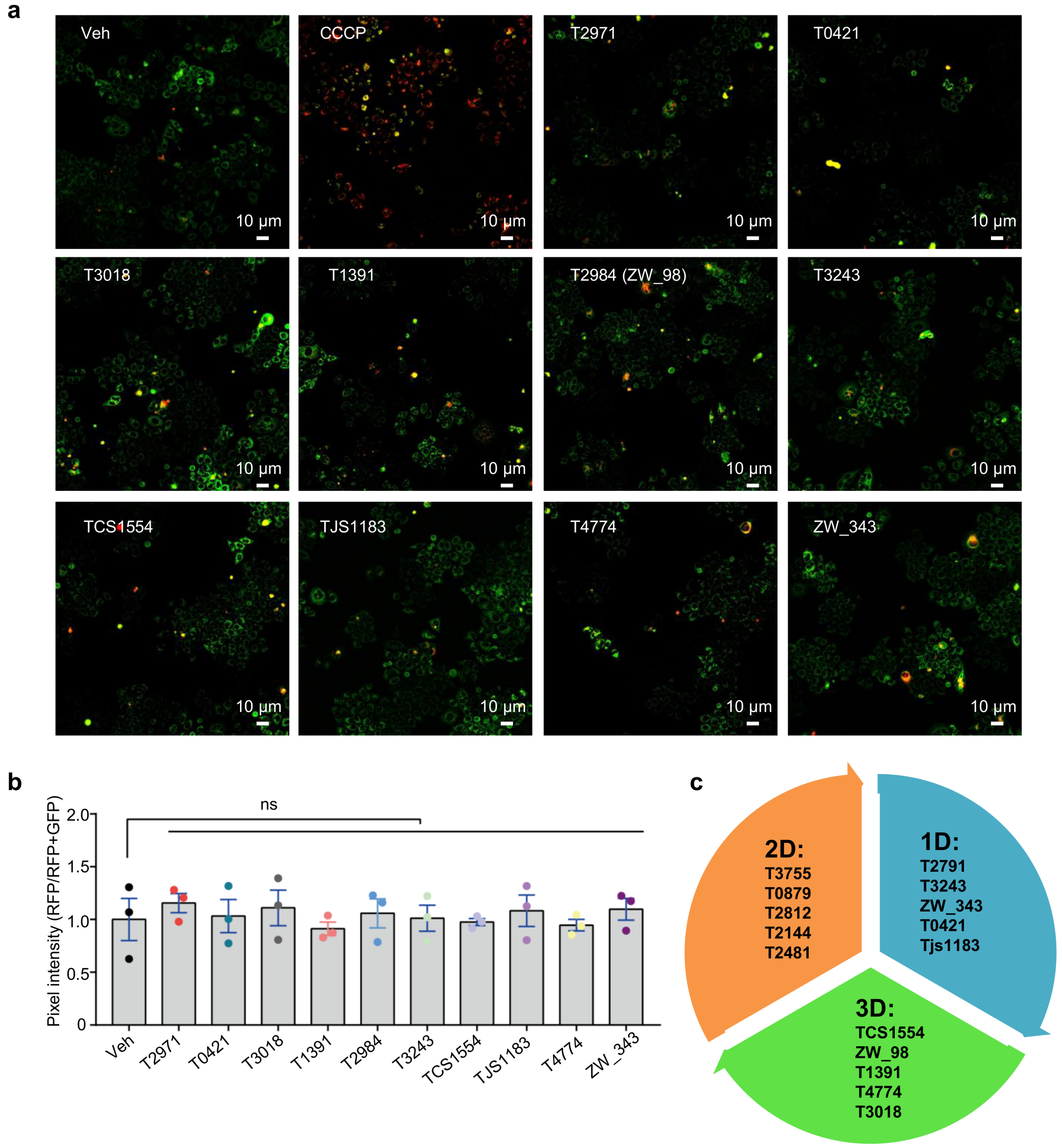

Supplementary Fig. 3 I Top 5 compounds selected by the 1D- or the 3D-AI model do not induce mitophagy in HeLa cells. a, Effects of designated compounds (10 µM for 24h) on mitophagy induction in HeLa-mt-Keima cells. **b,** Quantification of images from two biological repeats (with one representative set of images shown in 'a'). All quantitative data are shown in mean ± S.E.M. One-way ANOVA followed by Šidák's multiple comparisons test was used for data analysis. **c,** A list of the top 5 compounds selected by the 1D, 2D, or 3D AI model. For the 2D approach, all the top 5 compounds recommended were in the top 18 list selected by our combinational AI model: three compounds (T0879, T2812, T2144, at 10 μM) did not induce detectable mitophagy, the remaining two, ascomycin (T2481) and pinostilbene (T3755) were able to induce mitophagy in cells but were unable to induce neuronal mitophagy in nematodes (up to 1 mM, detailed in Fig. 2).

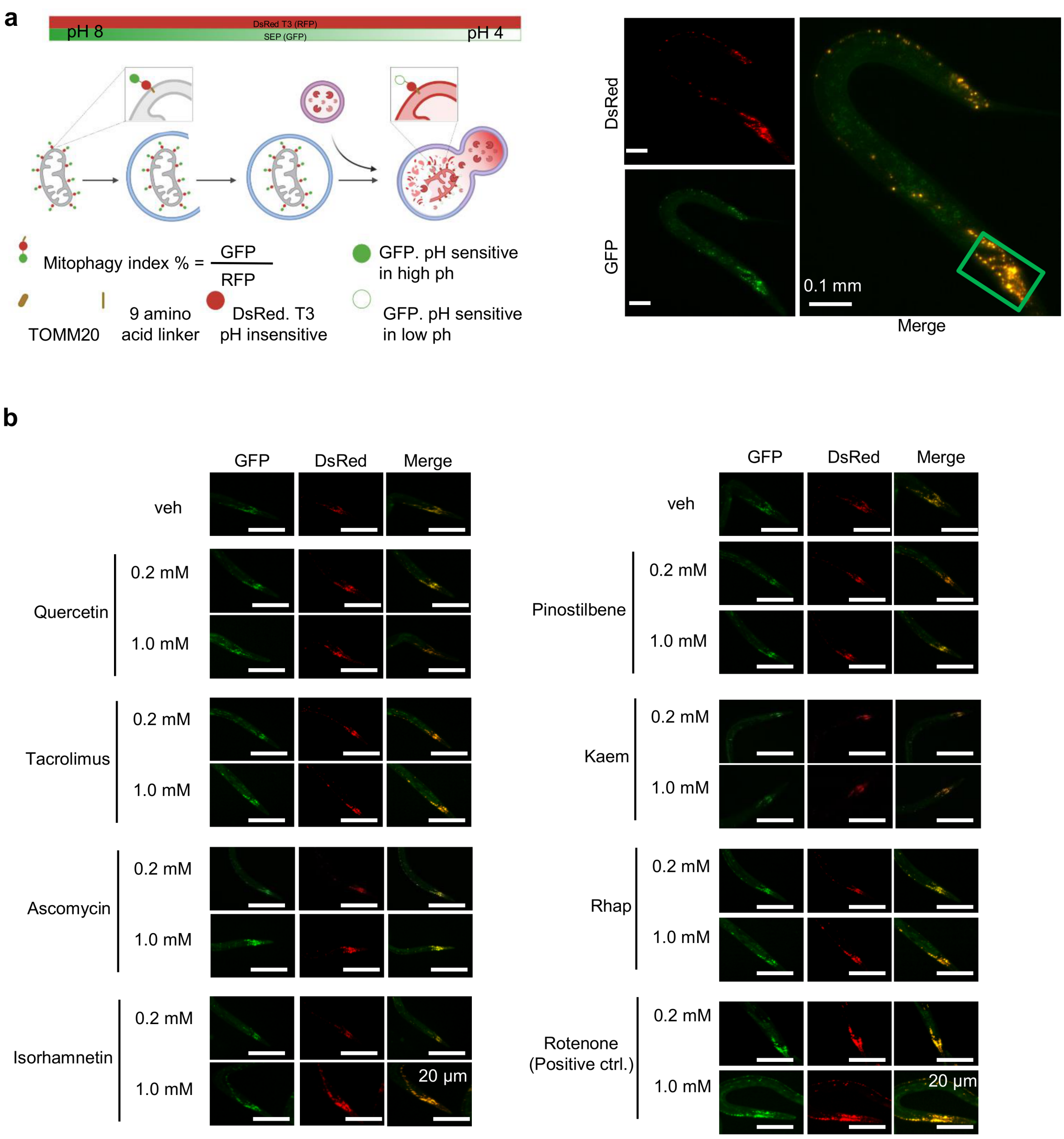

Supplementary Fig. 4 l Detection of *in vivo* mitophagy using mt-Rosella. a, Schematic working model of the mt-Rosella expressed in the pan-neuronal system of the *C. elegans* (Ex[$p_{unc\text{-}119}$TOMM-20::Rosella*; rol-6(su1006)*]). Mitophagy index is calculated as GFP/DsRed ratio, thus lower values correlate with higher mitophagy. A worm with enhanced neuronal mitophagy is shown on the right (the boxed area highlights head neurons). **b,** A representative set of images of Fig. 2d showing the levels of mitophagy observed within anterior head neurons of *C. elegans* following treatment with AI-selected candidates**.** Scale bar, 20 µm.

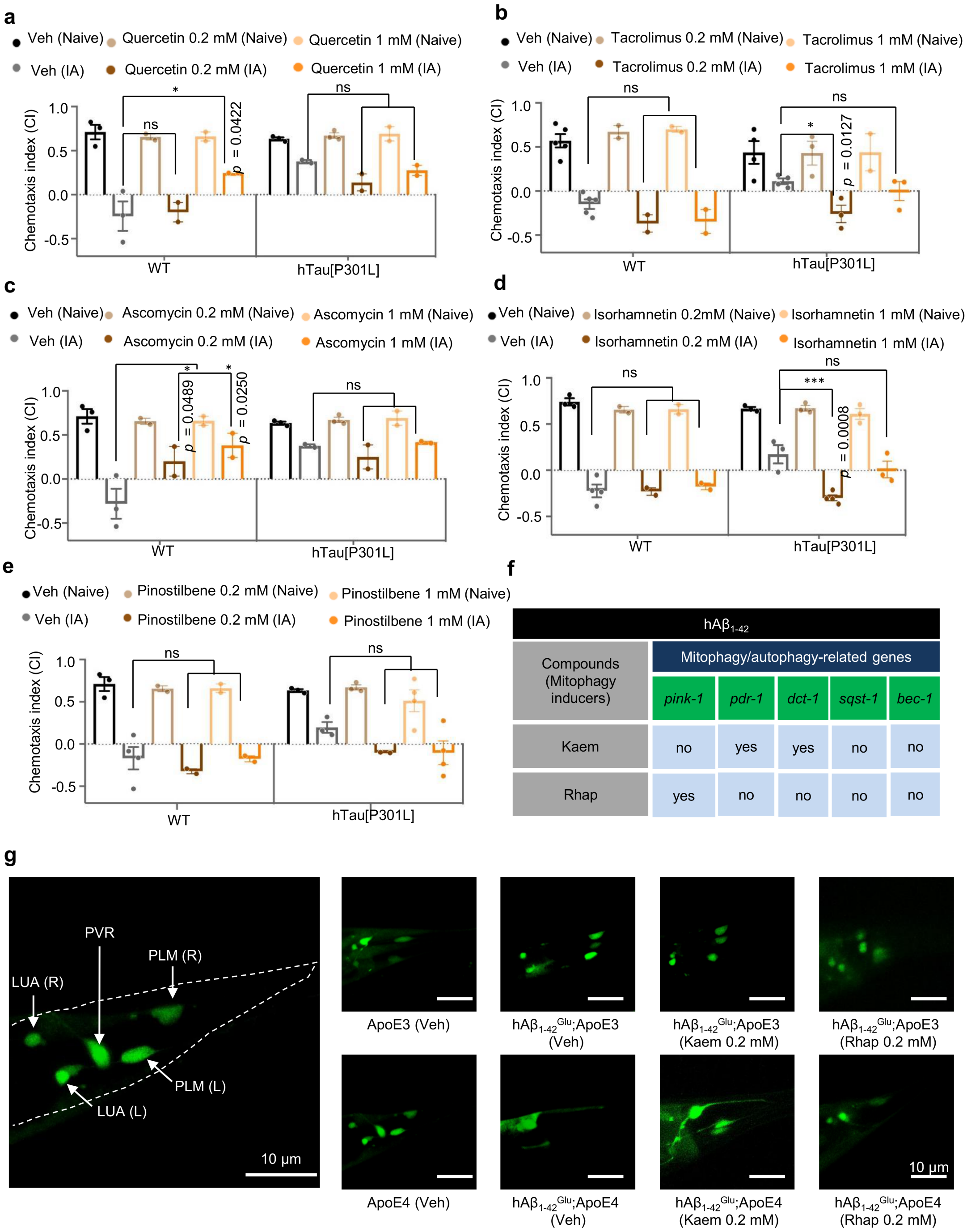

Supplementary Fig. 5 | Effects of different compounds on associative memory in the hTau[P301L] worms and on glutamatergic neuroprotection. a-e, Effects of Quercetin (**a**), Tacrolimus (**b**), Ascomycin (**c**), Isorhamnetin (**d**) and Pinosteilbene (**e**) on associative memory in transgenic nematodes expressing hTau[P301L] (CK12) at adult Day-1. All compounds were treated from egg hatching onwards. All quantitative data are shown in mean ± S.E.M from at least 3 biological replicates with 200-300 worms/group/biological repeat. Two-way ANOVA followed by Tukey's multiple comparisons test was used for data analysis. NS, no significance, $*p < 0.05$, $***p < 0.001$. **f**, A summary of mitophagy genes involved in Kaem and Rhap-induced memory improvement. **g**, Effects of Kaem or Rhap on glutamatergic neuroprotection in the hAβ $_{1\text{-}42}^{Glu}$; hApoE4^{Glu} worms and the other 7 worm strains as labelled. Left, a representative image showing the five designated tail neurons targeted for the assay. While there are approximately 15 *eat-4*-expressed tail neurons, the LUA (R), LUA (L), PVR, PLM (R), and PLM (L) neurons were scored within the posterior region of the animal as a mean to facilitate accurate quantification. Right, one representative image from each type of animal and the treatment used in the quantitative data analysis displayed in Fig. 4d. Scale bar, 10 μm. Also see Additional Supp. Video.

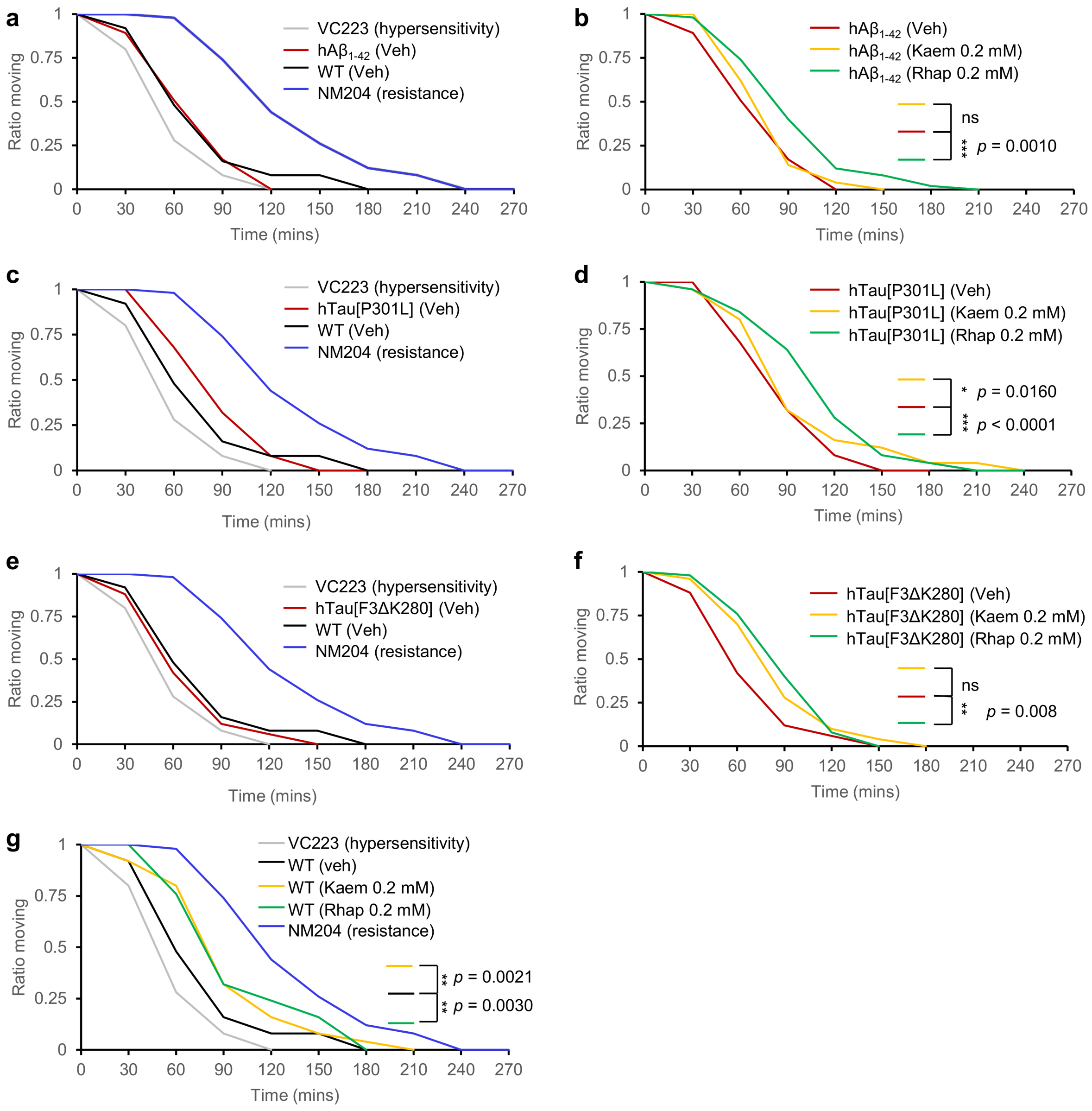

Supplementary Fig. 6 l Effects of Kaem or Rhap on the acetylcholinesterase inhibitor aldicarb-induced paralysis in both Aβ and Tau worms. a-g, Worm strains including hA$\beta_{1\text{-}42}$ (CL2355) (**a, b**), hTau[P301L] (CK12) (**c, d**), hTau[F3ΔK280] (BR5270) (**e, f**), and N2 (**g**) were examined following optimization of aldicarb concentration; 0.5 mM was chosen for the final experiments. VC223 (a strain hypersensitive to aldicarb-induced paralysis) and NM204 (a strain resistant to aldicarb-induced paralysis) were used as controls. Data were averaged from three biological replicates (30 worms/group/biological repeat). Parameters like mean, S.E.M., and p values were calculated using the log-rank test (Mantel-Cox), from a pooled population of animals. Quantified data shown in mean ± S.E.M are available in Supp. Table 5.

Supplementary Table 1 I List of the known mitophagy inducers as reference.

Mitophagy inducers	Formula	Molecular weight (g/mol)	Chemical structure
**#1. Rapamycin**	$C_{51}H_{79}NO_{13}$	914.172	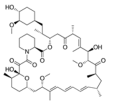
**#2. Pifithrin-a**	$C_{16}H_{19}BrN_2OS$	367.3	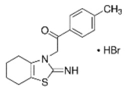
**#3. Deferiprone (DFP)**	$C_7H_9NO_2$	139.15	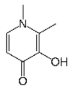
**#4. Metformin**	$C_4H_{11}N_5$	129.164	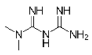
**#5. 1,10'-phenanthroline (Phen)**	$C_{12}H_8N_2$	180.21	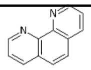
**#6. Ciclopirox olamine**	$C_{14}H_{24}N_2O_3$	268.35	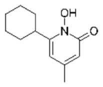
**#7. Nicotinamide riboside (NR)**	$C_{11}H_{15}N_2O_5+$	255.24	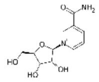
**#8. Actinonin**	$C_{19}H_{35}N_3O_5$	385.505	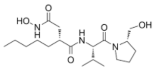
**#9. Nicotinamide mononucleotide (NMN)**	$C_{11}H_{15}N_2O_8P$	334.22	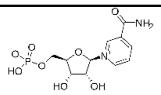
**#10. Resveratrol**	$C_{14}H_{12}O_3$	228.25	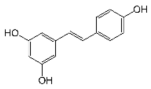
**#11. Fisetin**	$C_{15}H_{10}O_6$	286.236	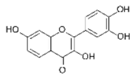
**#12. P62/SQSTM1-mediated mitophagy inducer (PMI)**	$C_{14}H_9IN_4O_2$	392.15	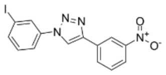
**#13. Spermidine**	$C_7H_{19}N_3$	145.25	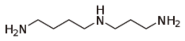
**#14. Urolithin A**	$C_{13}H_8O_4$	228.2	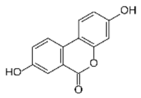

Supplementary Table 2 | Detailed information of the selected small compounds

Number	ID	Molename	CAS	Formula	MolWt	Source	Category
1	T2174	Quercetin	117-39-5	$C_{15}H_{10}O_7$	302.24	Plant	glycosides
2	T6630	Quercetin Dihydrate	6151-25-3	$C_{15}H_{10}O_7 \cdot 2H_2O$	338.27	Plant	Flavonoids
3	T2144	Tacrolimus	104987-11-3	$C_{44}H_{69}NO_{12}$	804.02	Microorganism	Others
4	T2481	Ascomycin	104987-12-4	$C_{43}H_{69}NO_{12}$	792.01	Microorganism	Others
5	T2836	Isorhamnetin	480-19-3	$C_{16}H_{12}O_7$	316.27	Plant	Flavonoids
6	T3755	Pinostilbene	42438-89-1	$C_{15}H_{14}O_3$	242.273	Plant	Phenols
7	T2177	Kaempferol (Kaem)	520-18-3	$C_{15}H_{10}O_6$	286.23	Plant	Flavonoids
8	T3776	Rhapontigenin (Rhap)	500-65-2	$C_{15}H_{14}O_4$	258.3	Plant	Phenols
9	T0579	Myricetin	529-44-2	$C_{15}H_{10}O_8$	318.24	Plant	Flavonoids
10	T2910	3-Hydroxyflavone	577-85-5	$C_{15}H_{10}O_3$	238.25	Animal	Flavonoids
11	T0610	Piceatannol	10083-24-6	$C_{14}H_{12}O_4$	244.24	Plant	Alcohol
12	T0879	Nicotinic acid	59-67-6	$C_6H_5NO_2$	123.11	Animal	Others
13	T3814	Isorhapontigenin	32507-66-7	$C_{15}H_{14}O_4$	258.273	Plant	Phenols
14	T3S1068	Oxyresveratrol	29700-22-9	$C_{14}H_{12}O_4$	244.24	Plant	Phenols
15	T7052	Gnetol	86361-55-9	$C_{14}H_{12}O_4$	244.24	Plant	Phenols
16	T3843	Pinosylvin	22139-77-1	$C_{14}H_{12}O_2$	212.248	Plant	Phenols
17	T2812	Adenosine disodium triphosphate	34369-07-8	$C_{10}H_{14}N_5Na_2O_{13}P_3$	551.15	Animal	Others
18	T1723	ADP	58-64-0	$C_{10}H_{15}N_5O_{10}P_2$	427.2	Organism	Others

Supplementary Table 3 I The hit rates for experimental HTS, computer aided, QSAR, and machine learning approaches.

**Approach (with PMID reference)**	**Hit rate**	**Data Size**	**Type**
Experimental HTS [PMID: 23975880]	0.01% to 0.14%	Not applicable	Traditional
Blue Dolphin - PTP-1B [PMID: 12014959]	4.93% (18/365)	250,000	Computer Aided
Blue Dolphin - AmpC β-lactamase [PMID: 12121656]	1.79% (1/56)	250,000	Computer Aided
Identification of Metabotropic Glutamate Receptor Subtype 5 Potentiators Using Virtual High-Throughput Screening [PMID: 20414370]	28.2%	450,000	QSAR
Discovery of New Anti-Schistosomal Hits by Integration of QSAR-Based Virtual Screening and High Content Screening [PMCID: PMC5844225]	6.90% (2/29)	150,000	QSAR
Generative tensorial reinforcement learning [PMID: 31477924]	15.00% (6/40)	30,000	Machine learning
Avalanche [PMID:30107123]	18.75% (6/32)	1,500,000	Machine learning
D-COID [PMID:32669436]	43.40% (10/23)	20,213	Machine learning
Machine learning. Virtual Screening and Molecular Docking [PMID:31167344]	8.96% (6/67)	1,457	Machine learning
Our Model (*in vitro*)	44.44% (8/18)	3,274	Machine learning

Supplementary Table 4 l A summary of lifespan values under different conditions.

**Experiments**	**Groups**	**Median lifespan (day)**	**Mean ± S.E.M. (Day)**	**Statistics (p values)**
Lifespan 1st repeat	N2 (Veh)	13	12.71 ± 0.26	
	N2 (Kaem 0.2 mM)	16	15.15 ± 0.32	p < 0.0001 vs. N2
	N2 (Rhap 0.2 mM)	17	16.84 ± 0.56	p < 0.0001 vs. N2
	N2 (Kaem 0.2 mM+Rhap 0.2 mM)	16	15.99 ± 0.60	p < 0.0001 vs. N2
	hTau[P301L] (Veh)	10	9.42 ± 0.28	p < 0.0001 vs. N2
	hTau[P301L] (Kaem 0.2 mM)	11	9.97 ± 0.31	p = 0.0019 vs. hTau[P301L] (Veh)
	hTau[P301L] (Rhap 0.2 mM)	10	11.06 ± 0.34	p = 0.0001 vs. hTau[P301L] (Veh)
	hTau[P301L] (Kaem 0.2 mM+Rhap 0.2 mM)	10	11.32 ± 0.44	p = 0.0016 vs. hTau[P301L] (Veh)
Lifespan 2nd repeat	N2 (Veh)	15	14.73 ± 0.39	
	N2 (Kaem 0.2 mM)	17	16.69 ± 0.30	p = 0.0025 vs. N2
	N2 (Rhap 0.2 mM)	20	19.39 ± 0.23	p < 0.0001 vs. N2
	N2 (Kaem 0.2 mM+Rhap 0.2 mM)	19	19.38 ± 0.18	p < 0.0001 vs N2<br>p = 0.3673 vs N2 (Rhap 0.2 mM)
	hTau[P301L] (Veh)	11	11.47 ± 0.33	p < 0.0001 vs. N2
	hTau[P301L] (Kaem 0.2 mM)	12	12.53 ± 0.36	p = 0.0018 vs. hTau[P301L] (Veh)
	hTau[P301L] (Rhap 0.2 mM)	18	17.04 ± 0.31	p < 0.0001 vs. hTau[P301L] (Veh)
	hTau[P301L] (Kaem 0.2 mM+Rhap 0.2 mM)	19	19.21 ± 0.32	p < 0.0001 vs. hTau[P301L] (Veh)<br>p < 0.0001 vs. hTau[P301L] (Rha 0.2 mM)

Supplementary Table 5 | Quantified data showing the effects of Kaem or Rhap on the acetylcholinesterase inhibitor aldicarb-induced paralysis in both Aβ and Tau worms.

**Groups**	**Median (min)**	**Mean ± S.E.M. (min)**	**Statistics (p values)**
VC223 (hypersensitivity) (Veh)	60	60.00 ± 2.89	
N2 (Veh)	90	82.40 ± 4.15	
N2 (Kaem 0.2 mM)	90	101.2 ± 5.06	$p = 0.0021$ vs. N2 (Veh)
N2 (Rhap 0.2 mM)	90	103.2± 5.17	$p = 0.0030$ vs. N2 (Veh)
hTau[P301L] (Veh)	90	92.80 ± 3.61	
hTau[P301L] (Kaem 0.2 mM)	90	105.2 ± 4.67	$p = 0.0160$ vs. hTau[P301L] (Veh)
hTau[P301L] (Rhap 0.2 mM)	120	116.8 ± 4.95	$p < 0.0001$ vs. hTau[P301L] (Veh)
hTau[F3ΔK280] (Veh)	60	74.40 ± 4.22	
hTau[F3ΔK280] (Kaem 0.2 mM)	90	91.80 ± 4.49	$p = 0.082$ vs. hTau[F3ΔK280] (Veh)
hTau[F3ΔK280] (Rhap 0.2 mM)	90	96.60 ± 4.047	$p = 0.008$ vs. hTau[F3ΔK280] (Veh)
hA$\beta_{1\text{-}42}$ (Veh)	90	77.23 ± 3.95	
hA$\beta_{1\text{-}42}$ (Kaem 0.2 mM)	90	84.00 ± 3.32	
hA$\beta_{1\text{-}42}$ (Rhap 0.2 mM)	90	100.2 ± 5.26	$p = 0.0010$ vs. hA$\beta_{1\text{-}42}$ (Veh)
NM204 (resistance) (Veh)	120	139.8 ± 4.87	

Supplementary Table 6 I List of worm strains used.

**Strain genotypes**	**Source**
N2: wild-type Bristol isolate	CGC
CK12: *Is[p*$_{aex\text{-}3}$tau4R1N(P301L); *p*$_{myo\text{-}2}$GFP*]*	Steven Zuryn lab
BR5270: *byIs161[p*$_{rab\text{-}3}$F3ΔK280*; p*$_{myo\text{-}2}$mCherry*]*	CGC
CL2355: *dvIs50[p*$_{snb\text{-}1}$Abeta$_{1\text{–}42}$*; p*$_{mtl\text{-}2}$GFP*]*	CGC
pink-1(tm1779)II; byIs161[p$_{rab\text{-}3}$F3ΔK280*;p*$_{myo\text{-}2}$mCherry*]*	Tavernarakis lab
pdr-1(gk448)III; byIs161[p$_{rab\text{-}3}$F3ΔK280*;p*$_{myo\text{-}2}$mCherry*]*	Tavernarakis lab
dct-1(tm376)X; byIs161[p$_{rab\text{-}3}$F3ΔK280*;p*$_{myo\text{-}2}$mCherry*]*	Tavernarakis lab
UA353: *baIn51[p*$_{eat\text{-}4}$*ApoE3; p*$_{unc\text{-}54}$*tdTomato];baIn34[p*$_{eat\text{-}4}$Abeta$_{1\text{-}42}$*; p*$_{myo\text{-}2}$mCherry*]; adIs1240[p*$_{eat\text{-}4}$GFP*]*	Caldwell lab
UA355: *baIn52[p*$_{eat\text{-}4}$*ApoE4; p*$_{unc\text{-}54}$*tdTomato];baIn34[p*$_{eat\text{-}4}$Abeta$_{1\text{-}42}$*; p*$_{myo\text{-}2}$mCherry*]; adIs1240[p*$_{eat\text{-}4}$GFP*]*	Caldwell lab
UA357: *baIn52[p*$_{eat\text{-}4}$*ApoE3;p*$_{unc\text{-}54}$*tdTomato];adIs1240[p*$_{eat\text{-}4}$GFP*]*	Caldwell lab
UA358: *baIn53[p*$_{eat\text{-}4}$*ApoE4;p*$_{unc\text{-}54}$*tdTomato];adIs1240[p*$_{eat\text{-}4}$GFP*]*	Caldwell lab
neuronal mt-Rosella: *N2; Ex[p*$_{unc\text{-}119}$TOMM-20*::Rosella; rol-6(su1006)]*	Tavernarakis lab
VC223: *tom-1(ok285)I*	CGC
NM204: *snt-1(md290)II*	CGC
IR2160: *N2; Ex002[Prab-3::dsRed::LGG- 1Pprab-3DCT-1::gfp; Pmyo-2::gfp]*	Tavernarakis Lab
EFF060: *dvIs50 [pCL45 (snb-1::Abeta 1-42::3' UTR(long) + mtl-2::*GFP*];sid-1(pk3321) V;uIs69 [pCFJ90 (myo-2p::*mCherry*) + unc-119p::sid-1] V*	The Fang Lab
EFF029: *Is[paex-3::*tau4R1N(P301L) *+ pmyo-2::gfp];sid-1(pk3321) V;uIs69 [pCFJ90 (myo-2p::mCherry) + unc-119p::sid-1] V*	The Fang Lab

Additional Supplementary Information

Supplementary Dataset I Analysis of chemical similarities of the top 18 AI-suggested compounds (Microsoft Excel file).

Supplementary Video 1 I One representative 3-D image showing the five designated tail neurons targeted for the assay. They were LUA (R), LUA (L), PVR, PLM (R), and PLM (L) neurons. Also see Supp. Fig.5.
