# Supplementary material for: Amelioration of Alzheimer’s disease pathology by mitophagy inducers identified via machine learning and a cross-species workflow
Source: Nat Biomed Eng. 2022 Jan 6;6(1):76–93. doi: 10.1038/s41551-021-00819-5 (PMC8782726; doi:10.1038/s41551-021-00819-5)
Supplement: Supplementary file 2 — Reporting Summary [file 41551_2021_819_MOESM2_ESM.pdf]

nature portfolio

Corresponding author(s): Evandro F. Fang and Jia-Hong Lu

Last updated by author(s): Sep 15, 2021

Reporting Summary

Nature Portfolio wishes to improve the reproducibility of the work that we publish. This form provides structure for consistency and transparency in reporting. For further information on Nature Portfolio policies, see our Editorial Policies and the Editorial Policy Checklist.

Statistics

For all statistical analyses, confirm that the following items are present in the figure legend, table legend, main text, or Methods section.

n/a	Confirmed	
☐	☒	The exact sample size (n) for each experimental group/condition, given as a discrete number and unit of measurement
☐	☒	A statement on whether measurements were taken from distinct samples or whether the same sample was measured repeatedly
☐	☒	The statistical test(s) used AND whether they are one- or two-sided<br>*Only common tests should be described solely by name; describe more complex techniques in the Methods section.*
☒	☐	A description of all covariates tested
☒	☐	A description of any assumptions or corrections, such as tests of normality and adjustment for multiple comparisons
☐	☒	A full description of the statistical parameters including central tendency (e.g. means) or other basic estimates (e.g. regression coefficient) AND variation (e.g. standard deviation) or associated estimates of uncertainty (e.g. confidence intervals)
☐	☒	For null hypothesis testing, the test statistic (e.g. F, t, r) with confidence intervals, effect sizes, degrees of freedom and P value noted<br>*Give P values as exact values whenever suitable.*
☒	☐	For Bayesian analysis, information on the choice of priors and Markov chain Monte Carlo settings
☒	☐	For hierarchical and complex designs, identification of the appropriate level for tests and full reporting of outcomes
☐	☒	Estimates of effect sizes (e.g. Cohen's d, Pearson's r), indicating how they were calculated

Our web collection on statistics for biologists contains articles on many of the points above.

Software and code

Policy information about availability of computer code

Data collection	RDkit-2019 was used for molecule operation and similarity calculation. Scikit-learn 0.23 was used for data processing and clustering.<br>Custom code was specific to our computing infrastructure and mainly used for data input/output and parallelization across computers and graphics processors. The code, currently under a patent-examination process, is available from the corresponding author on reasonable request.<br>Confocal Labmaze was used for behavioural tracking in the mouse studies.<br>Nikon, ZEN (blue edition) and Leica TCS SP8 confocal Laser Scanning Microscope System software were used for taking images.
Data analysis	Microsoft Excel (2016) and Graphpad Prism 8 were used for data analysis. ImageJ with the plug in ObjectJ was used for the quantification of mitophagy levels.

For manuscripts utilizing custom algorithms or software that are central to the research but not yet described in published literature, software must be made available to editors and reviewers. We strongly encourage code deposition in a community repository (e.g. GitHub). See the Nature Portfolio guidelines for submitting code & software for further information.

Data

Policy information about availability of data

All manuscripts must include a data availability statement. This statement should provide the following information, where applicable:

- Accession codes, unique identifiers, or web links for publicly available datasets
- A description of any restrictions on data availability
- For clinical datasets or third party data, please ensure that the statement adheres to our policy

The main data supporting the findings of this study are available within the Article and its Supplementary Information. The raw wet-lab data generated in this study

are available from the corresponding author on reasonable request. Restrictions apply to the AI-related data: all requests for raw and processed data will be reviewed by Mindrank AI to verify whether the request is subject to any intellectual property or confidentiality constraints.

Field-specific reporting

Please select the one below that is the best fit for your research. If you are not sure, read the appropriate sections before making your selection.

☒ Life sciences ☐ Behavioural & social sciences ☐ Ecological, evolutionary & environmental sciences

For a reference copy of the document with all sections, see nature.com/documents/nr-reporting-summary-flat.pdf

Life sciences study design

All studies must disclose on these points even when the disclosure is negative.

Sample size	Sample sizes for the behavioral tests were determined by the current standard used for mice in behavioral experiments, as well as based on the minimal amount of mice required to detect statistical significance with an alpha rate set at 0.05 in a standardly powered experiment. Thus, a total 6 mice/group were used for behavioral tests. The sample sizes used for immuno-histochemistry, Western blotting and other analyses were 3–6, as specified in the figure captions. Sample sizes for the C. elegans studies were 200–400 worms/group for the memory assay and around 100 worms/group for the lifespan studies. The sample sizes used in other experiments are detailed in the respective figure captions.
Data exclusions	No data were excluded from analysis.
Replication	All the cell-culture and C. elegans experiments were performed with 3 biological repeats (with 3 technical repeats within each biological repeat), unless otherwise specified. All experiments were replicated successfully, with consistent data. The mouse data were from one biological experiment, as is common in the field.
Randomization	Animals and samples were assigned randomly to the various experimental groups. Mice were randomly selected for the behavioural experiments.
Blinding	In data collection and analysis (mouse behavioural studies, mouse imaging-data analysis, and the imaging and data analysis of electron microscopy), the investigators were blinded to the experimental design.

Reporting for specific materials, systems and methods

We require information from authors about some types of materials, experimental systems and methods used in many studies. Here, indicate whether each material, system or method listed is relevant to your study. If you are not sure if a list item applies to your research, read the appropriate section before selecting a response.

Materials & experimental systems

n/a	Involved in the study	
☐	☒	Antibodies
☐	☒	Eukaryotic cell lines
☒	☐	Palaeontology and archaeology
☐	☒	Animals and other organisms
☒	☐	Human research participants
☒	☐	Clinical data
☒	☐	Dual use research of concern

Methods

n/a	Involved in the study	
☒	☐	ChIP-seq
☒	☐	Flow cytometry
☒	☐	MRI-based neuroimaging

Antibodies

Antibodies used	Antibodies used in western blot as follows (all from Cell Signaling Technology unless otherwise stated): PINK1 antibody (catalog no. ab75487, Abcam; no. A7131, ABclonal); Parkin antibody (no.NB100-91921; Novus); FUNDC1 antibody (no. ab74834; Abcam); LC3B antibody (no.NB100-2220; Novus); Beclin1 antibody (no.3495s); phospho-DRP1 antibody (no.S616); DRP1 antibody (no.8570s); p62 antibody (no.8025s); MFN2 antibody (no.94823s); phospho-ULK1 antibody (no.5869s); ULK1 antibody (no.6439s); AMBRA1 antibody (no.24907s); OPTN antibody (no. A1845, ABclona); Tau antibody (no. 46687s); p-Tau-thr181 (no. 12285s); p-Tau-thr231 (no. ab151559, Abcam); p-Tau-ser202/thr205 (no. MN1020, ThermoFisher Scientific); p-Tau-thr217 (no. 44-744, ThermoFisher Scientific); beta Amyloid polyclonal antibody (no. 51-2700, ThermoFisher Scientific); β-actin antibody (no. A5441; Sigma). Secondary antibodies including anti-mouse immunoglobulin G (IgG; catalog no. 7076s) and anti-rabbit IgG (no. 7074s).<br>Specific primary antibodies used in immunohistochemistry include: mouse anti-β-Amyloid, 1-16 antibody (clone 6E10, catalog no. 803002; BioLegend); and rabbit anti-iba1 antibody (019-19741; Wako); mouse anti-AT8 antibody (cat no. MN1020; ThermoFisher Scientific). For first antibodies, the dilution ratio was 1:1000 or otherwise as specified else where; for secondary antibodies, the dilution ration was 1:5000 or otherwise as specified else where.
Validation	All the antibodies were validated for use in cell, mouse/human tissues based on previous publications (Fang EF et al., Cell 2014; Fang

Validation	EF et al., Cell Metab 2016; Fang EF et al., Nat Neuroscu 2019; Fang EF et al., Nat Commun 2019). Detailed antibody validation profiles are available on the antibody-provider websites.

Eukaryotic cell lines

Policy information about cell lines

Cell line source(s)	Mt-Keima HeLa cells were used for mitophagy evaluation; HEK293 cells stably expressing the 0N4R isoform of human Tau, bearing the P301S mutation with a C-terminal venus were used for Tau aggregation and degradation. N2a and GFP-LC3 HeLa cells were cultured for mechanistic studies.
Authentication	None of the cell lines used were authenticated.
Mycoplasma contamination	All cell lines tested negative for mycoplasma contamination.
Commonly misidentified lines (See ICLAC register)	No commonly misidentified cell lines were used.

Animals and other organisms

Policy information about studies involving animals; ARRIVE guidelines recommended for reporting animal research

Laboratory animals	3xTg-AD mice were provided on a B6;129 genetic background (Stock No. 004807 B6;129-Tg(APPSwe,tauP301L)1Lfa Psen1tm1Mpm/Mmjax; https://www.jax.org/strain/004807) by the Jackson's laboratory. C57BL/6J mice were used as WT control. All animals were housed in individually ventilated cages on standardized rodent bedding. All animals were housed under constant-light cycle (12 h light/dark) with food and water provided. 12.5-month-old 3xTg-AD female mice were used for two-month drug treatments by oral gavage, followed by behavioural and biochemical studies.
Wild animals	The study did not involve wild animals.
Field-collected samples	The study did not involve samples collected from the field.
Ethics oversight	All animal care and experimental procedures were approved by the Committee on the Ethics of Animal Experiments of the University of Macau (UMARE-013-2019).

Note that full information on the approval of the study protocol must also be provided in the manuscript.
