# Supplementary figures and images for: Amelioration of Alzheimer’s disease pathology by mitophagy inducers identified via machine learning and a cross-species workflow
Source: Nat Biomed Eng. 2022 Jan 6;6(1):76–93. doi: 10.1038/s41551-021-00819-5 (PMC8782726; doi:10.1038/s41551-021-00819-5)

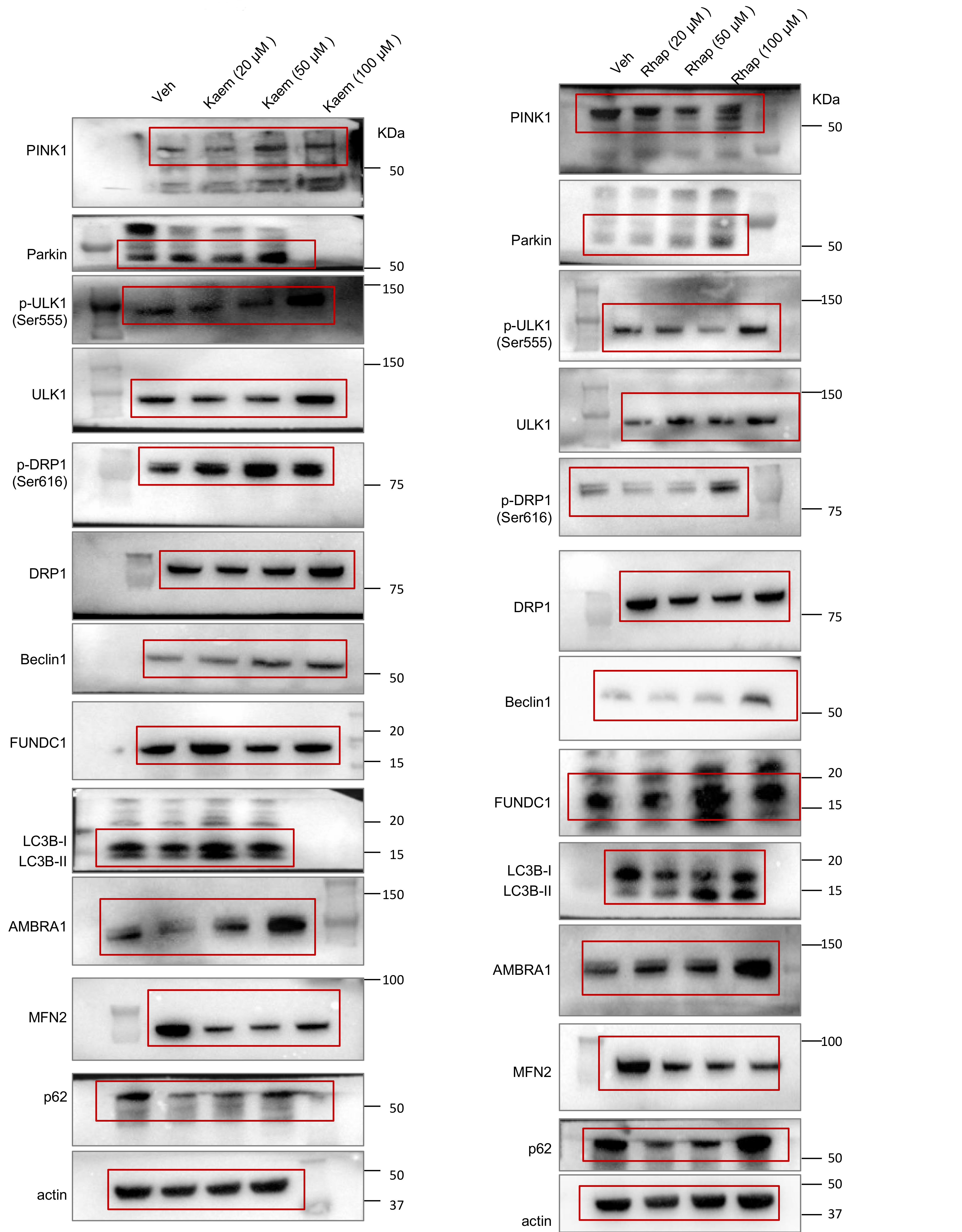

Supplement: Source Data Fig. 1 — Unprocessed western blots for Fig. 2a. [file 41551_2021_819_MOESM5_ESM.tiff]

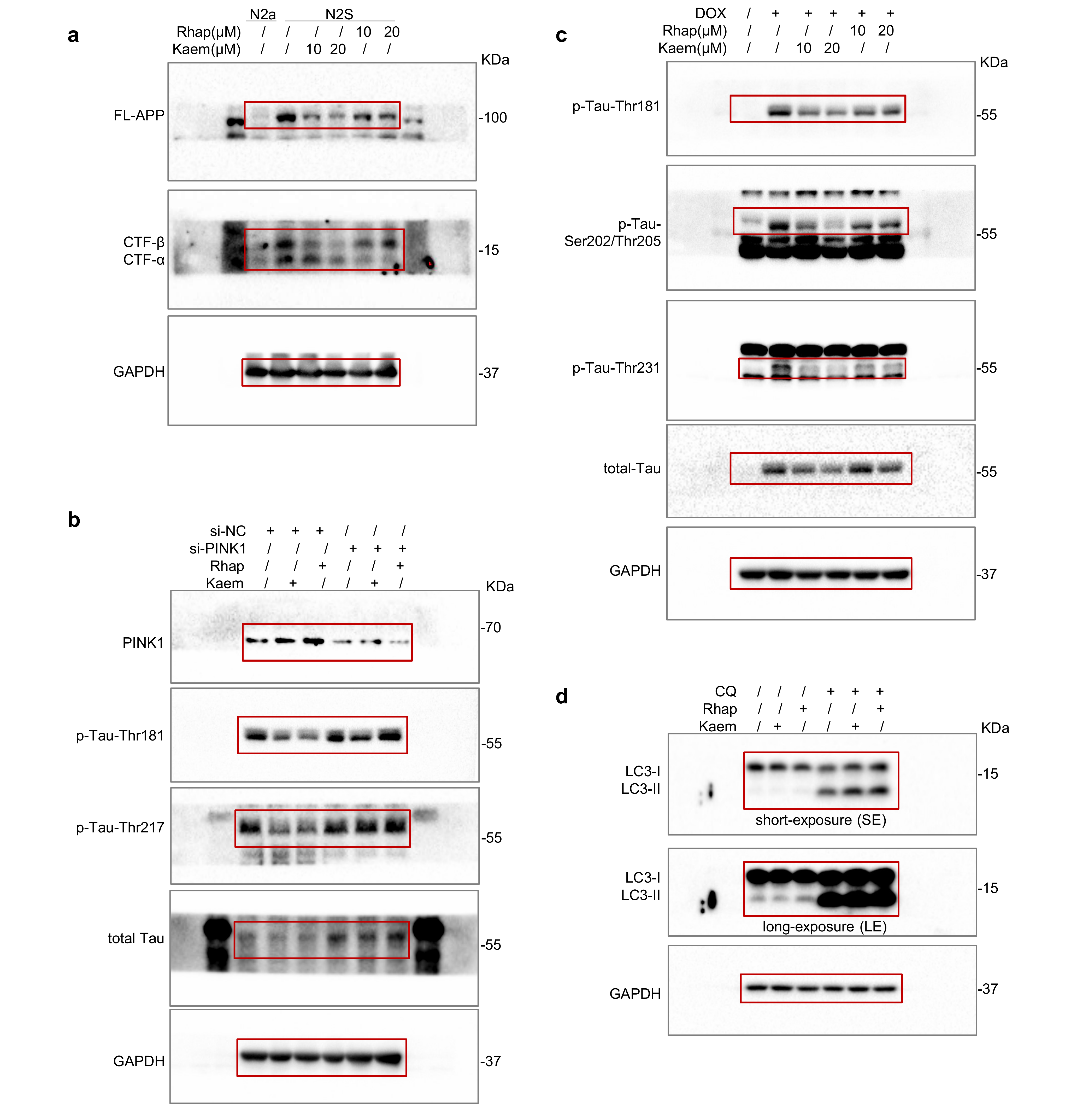

Supplement: Source Data Fig. 2 — Unprocessed western blots corresponding to Fig. 5f and Extended Data Figs. 1c,m and 4o. [file 41551_2021_819_MOESM6_ESM.tiff]

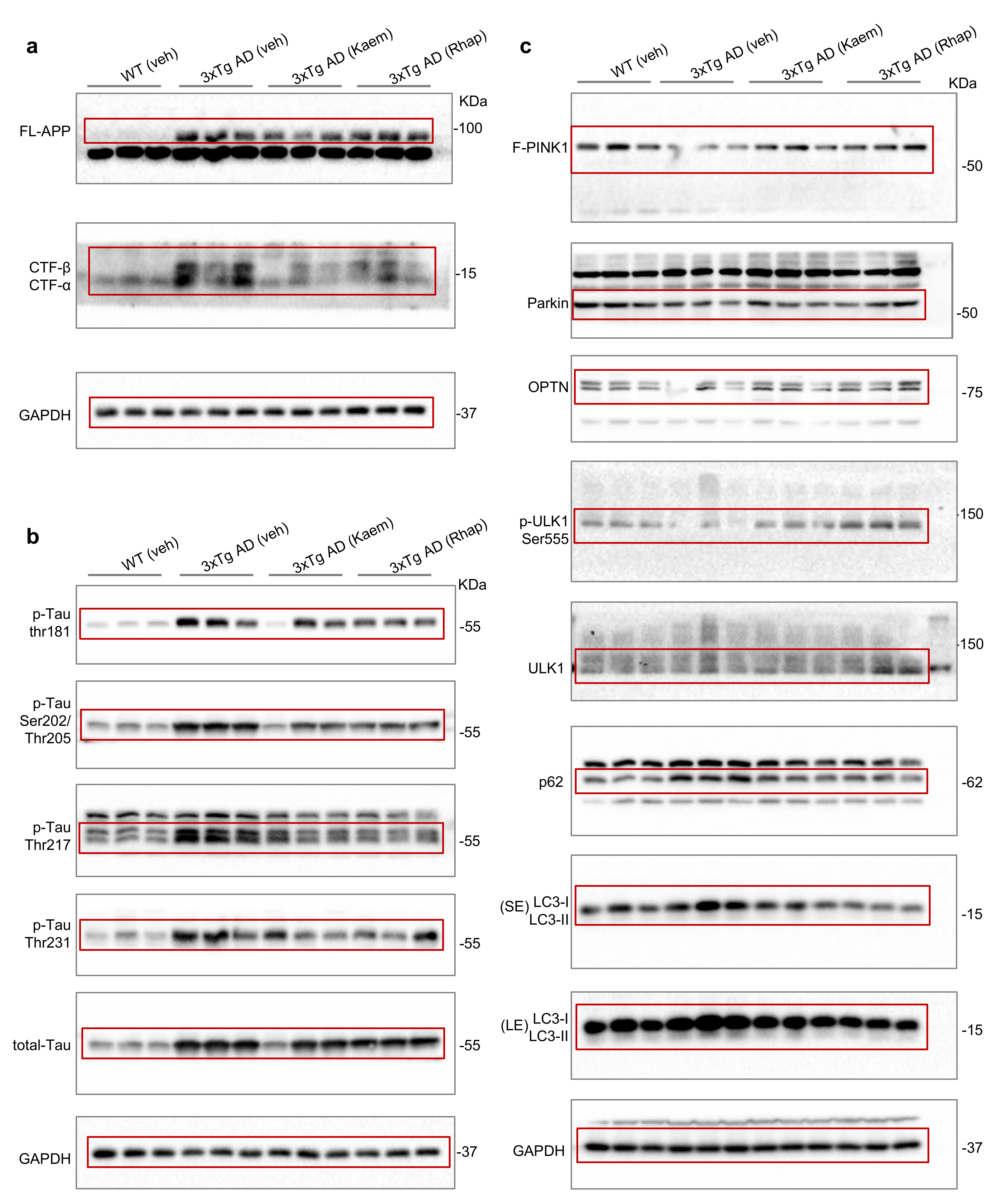

Supplement: Source Data Fig. 3 — Unprocessed western blots corresponding to Fig. 6o and Extended Data Fig. 4a,f. [file 41551_2021_819_MOESM7_ESM.tiff]

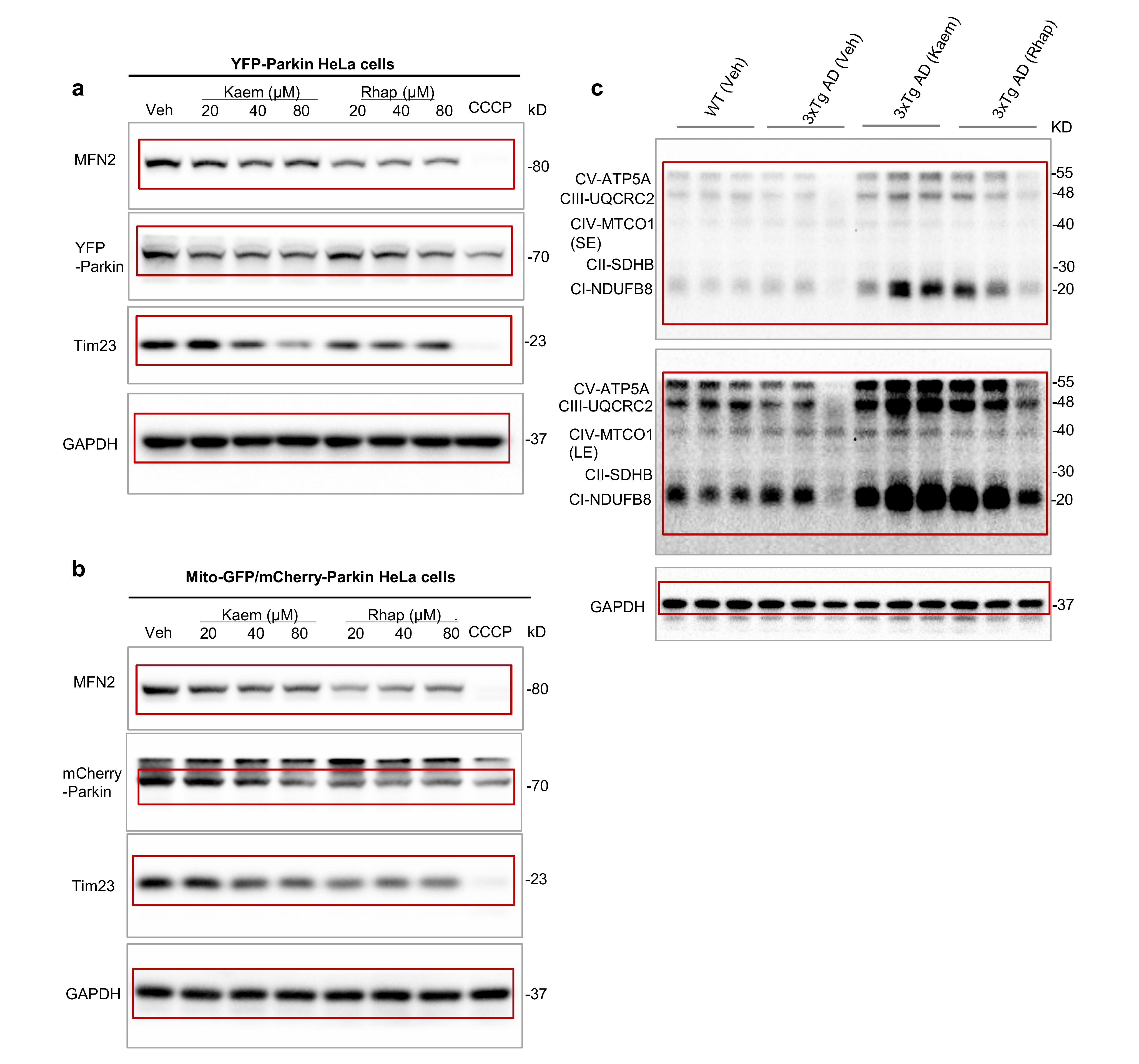

Supplement: Source Data Fig. 4 — Unprocessed western blots corresponding to Fig. 3a,e and Extended Data Fig. 4m. [file 41551_2021_819_MOESM8_ESM.tiff]
